# Drug-induced oxidative stress actively prevents caspase activation and hepatocyte apoptosis

**DOI:** 10.1038/s41419-024-06998-8

**Published:** 2024-09-09

**Authors:** Rebekka Lambrecht, Jasmin Jansen, Franziska Rudolf, Mohamed El-Mesery, Sabrina Caporali, Ivano Amelio, Florian Stengel, Thomas Brunner

**Affiliations:** 1 Biochemical Pharmacology, Department of Biology, University of Konstanz, Konstanz, Germany; 2 Biochemistry and Mass Spectrometry, Department of Biology, University of Konstanz, Konstanz, Germany; 3 Konstanz Research School Chemical Biology, University of Konstanz, Konstanz, Germany; 4 Collaborative Research Center TRR 353, Konstanz, Germany; 5 Department of Biochemistry, Faculty of Pharmacy, Mansoura University, Mansoura, Egypt; 6 Systems Toxicology, Department of Biology, University of Konstanz, Konstanz, Germany; 7 Present Address: Systems Immunology, Weizmann Institute of Science, Rehovot, Israel

**Keywords:** Proteases, Preclinical research

## Abstract

Cell death is a fundamental process in health and disease. Emerging research shows the existence of numerous distinct cell death modalities with similar and intertwined signaling pathways, but resulting in different cellular outcomes, raising the need to understand the decision-making steps during cell death signaling. Paracetamol (Acetaminophen, APAP)-induced hepatocyte death includes several apoptotic processes but eventually is executed by oncotic necrosis without any caspase activation. Here, we studied this paradoxical form of cell death and revealed that APAP not only fails to activate caspases but also strongly impedes their activation upon classical apoptosis induction, thereby shifting apoptosis to necrosis. While APAP intoxication results in massive drop in mitochondrial respiration, low cellular ATP levels could be excluded as an underlying cause of missing apoptosome formation and caspase activation. In contrast, we identified oxidative stress as a key factor in APAP-induced caspase inhibition. Importantly, caspase inhibition and the associated switch from apoptotic to necrotic cell death was reversible through the administration of antioxidants. Thus, exemplified by APAP-induced cell death, our study stresses that cellular redox status is a critical component in the decision-making between apoptotic and necrotic cell death, as it directly affects caspase activity.

## Introduction

Cell death is a fundamental process involved in several physiological processes, but also contributes to tissue damage during the pathogenesis of several diseases. In the past decades, numerous different forms of programmed cell death (PCD) have been discovered, and emerging research further emphasized their entanglement [1]. Thus, it is pivotal to fully understand the molecular complexity of cell death and the putative intersection points of different cell death modes. Apoptosis is the most studied form of PCD and is characterized by a controlled and distinct dismantling of dying cells, which is orchestrated by specific proteases, called caspases [2, 3]. In hepatocytes, extrinsic and intrinsic apoptotic stimuli are both integrated via the B cell lymphoma 2 (BCL-2) protein family at the mitochondria. BCL-2 proteins regulate mitochondrial outer membrane permeabilization (MOMP), one of the most critical steps during apoptosis [4]. MOMP results in the release of cytochrome c and SMAC, promoting of apoptosome formation and Caspase 9 activation, thereby initiating a self-amplifying caspase activation cascade and ultimately leading to apoptotic cell death [5, 6]. In marked contrast, classical necrosis is defined as the bursting of cells in response to overwhelming physical and chemical cellular stress without distinct preceding signaling events or a common morphology.

Paracetamol (Acetaminophen, APAP) is a widely used medication for pain and fever. Although safe at therapeutic doses, acute or cumulative overdose causes severe liver damage through massive hepatocyte necrosis [7]. In hepatocytes, APAP is metabolized to the toxic intermediate N-acetyl-p-benzoquinone imine (NAPQI), which forms covalent, irreversible adducts on proteins and DNA, resulting in multiple intracellular stress sites [8–11]. NAPQI is detoxified by cellular glutathione (GSH), however, once this detoxification pathway is saturated, the GSH pool rapidly drops, NAPQI accumulates, and hepatocytes face massive oxidative stress. APAP-induced reactive oxygen (ROS) and nitrogen species (RNS) severely damage mitochondria, resulting in a pronounced decline in mitochondrial energy production and eventually in a fatal self-amplifying loop of ROS and mitochondrial damage [12–17].

Despite extensive research, the mode of APAP-induced cell death is still highly controversial [18–23]. Morphologically, hepatocytes die clearly by oncotic necrosis with cellular swelling and membrane rupture, but the detection of numerous signaling events typically associated with apoptosis caused a lot of confusion and often led to misleading conclusions. We have previously shown that APAP-treated hepatocytes strongly upregulate various pro-apoptotic BCL-2 homologs and experience MOMP, but fail to activate caspases and to undergo apoptotic cell death [17]. Here, we investigate in detail at which step and by which mechanism the apoptotic signaling is blocked and switched to necrotic cell death.

Although apoptosome formation requires cellular ATP, and APAP treatment of hepatocytes results in a massive drop in cellular ATP, we could exclude low ATP levels as the underlying reason for missing caspase activation. Similarly, we did not find evidence of NAPQI conjugation to caspases and associated inactivation. In contrast, we find that the massive oxidative stress upon APAP directly prevents caspase activation and redirects hepatocytes into necrosis, despite the preceding apoptosis-promoting signaling processes. Hereby, we reveal a reversible decision-making process between apoptotic and necrotic cell death that is determined by cellular redox status.

## Results

### APAP-induced apoptotic signaling halts after MOMP

**Fig. 1:**
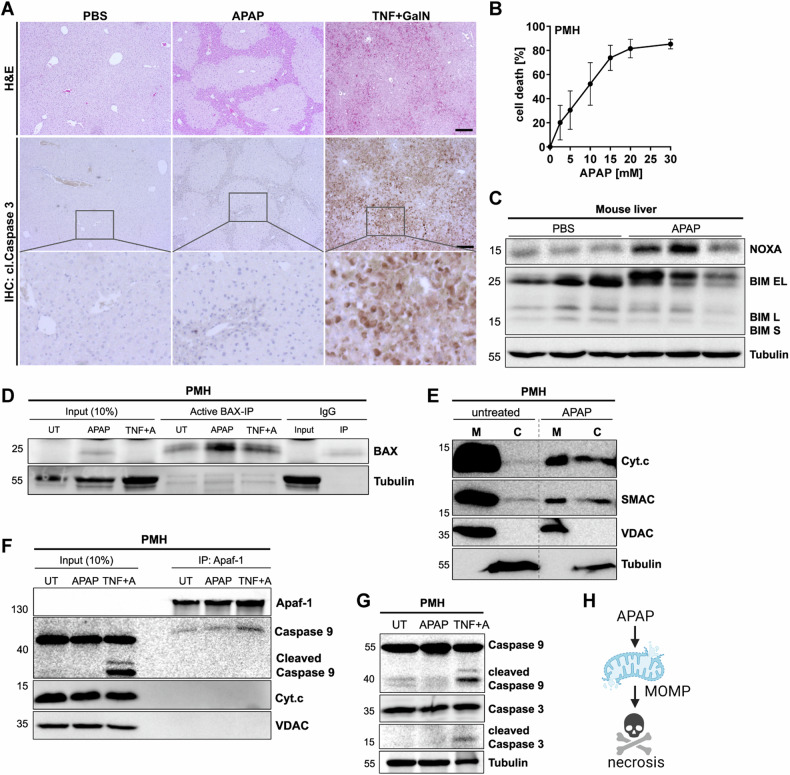
APAP-induced apoptotic signaling halts after MOMP. **A** Representative images of H&E- and anti-cleaved Caspase 3-stained liver sections of mice treated for 6 h either with PBS, 500 mg/kg APAP or 25 µg/kg murine TNF and 1 g/kg D-Galactosamine (GalN) i.p. Scale bar 150 µm. Lowest panel shows enlargement of selected areas. **B** MTT assay of primary murine hepatocytes (PMH) treated with indicated concentrations of APAP for 16 h. Mean ± SD of *n* = 3 independent experiments are shown. **C** Western Blot of murine whole liver lysates of mice treated with PBS or APAP as described in (**A**). Lanes represent samples from *n* = 3 mice per group. Numbers on the left indicate molecular weight in kDa. **D** Western Blot of active BAX (anti-NT-BAX) immunoprecipitation (IP) of PMH left untreated (UT) or treated with 20 mM APAP or 1 ng/ml TNF plus 50 nM Actinomycin D (TNF + A) for 3 h. **E** Western blot of mitochondrial (M) and cytosolic (C) fractions of PMH treated with 10 mM APAP for 6 h. **F** Western Blot of anti-Apaf-1 immunoprecipitation (IP) of PMH left untreated (UT) or treated with 20 mM APAP or 10 ng/ml TNF plus 30 nM Actinomycin D (TNF + A) for 6 h. **G** Western Blot of PMH left untreated (UT) or treated with 20 mM APAP or 10 ng/ml TNF plus 30 nM Actinomycin D for 12 h. **H** Schematic illustration summarizing that APAP treatment initiates apoptotic processes including MOMP in hepatocytes but fails to activate caspases and ultimately ends in necrosis.

The active, cleaved form of Caspase 3 represents one of the most important hallmarks of apoptosis [3] and can be observed during apoptosis-mediated liver damage in response to a wide range of stimuli. Accordingly, antibodies recognizing cleaved Caspase 3 can be used to detect apoptotic cell death in various tissues [24]. To study apoptotic and necrotic cell death induction, we treated mice with APAP or with the classical apoptosis inducer tumor necrosis factor (TNF) in combination with D-Galactosamine (GalN), and evaluated liver histology. Both, APAP and TNF plus GalN resulted in massive hepatocyte death with a typical zonal distribution in APAP-treated mice. Despite extensive liver damage in APAP-treated mice, no cleaved Caspase 3 staining was detected (Fig. 1A). In marked contrast, extensive Caspase 3 activation was detected in TNF plus GalN-treated mice. To further characterize APAP-induced cell death, primary murine hepatocytes (PMH) were treated ex vivo with APAP and examined. PMH showed dose-dependent cell death and the induction of the BH3-only proteins NOXA and BIM following APAP treatment (Fig. 1B, C). We have recently demonstrated that these pro-apoptotic BH3-only proteins also interact with their anti-apoptotic counterparts upon APAP treatment [17]. This interaction and the disbalance between anti- and pro-apoptotic BCL-2 proteins resulted in the activation of the pore-forming member BAX, which could be detected with an N-terminus-specific antibody (Fig. 1D). BAX activation promoted MOMP as evidenced by the release of cytochrome c and SMAC following APAP treatment (Fig. 1E). During classical apoptosis signaling, cytosolic cytochrome c initiates the formation of the heptameric apoptosome complex, composed of Apaf-1, pro-Caspase 9, cytochrome c and ATP as co-factor [25, 26]. In contrast to TNF, APAP failed to promote the binding of Caspase 9 to Apaf-1 (Fig. 1F). In line with this observation, Caspase 9 and Caspase 3 were not processed in response to APAP (Fig. 1G). These results were confirmed in the hepatocellular carcinoma cell line HepG2 (Fig. S1). In summary, apoptotic signaling appeared to be correctly initiated upon APAP treatment and successfully progressed until MOMP, but subsequently failed to activate caspases, ultimately resulting in hepatocyte necrosis (Fig. 1H).

### APAP actively prevents caspase activation upon classical apoptosis induction

**Fig. 2:**
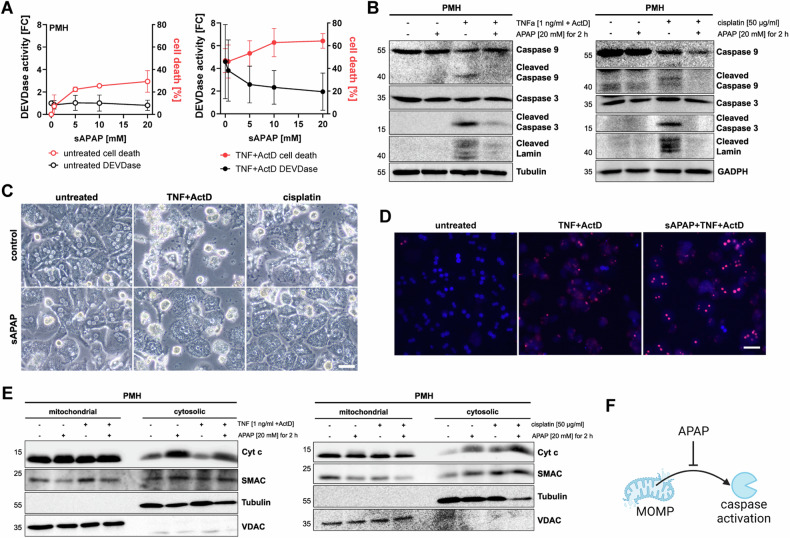
APAP actively prevents caspase activation during classical apoptosis. **A** Primary murine hepatocytes (PMH) were treated for 2 h with indicated concentrations of APAP. Then, APAP was removed (short APAP, sAPAP), and PMH were either left untreated (left graph) or treated with 2 ng/ml TNF plus 30 nM ActD for 16 h (right graph). Results of the MTT (red) and DEVDase assay (black) are shown as mean ± SD of *n* = 4. **B** PMH were pretreated with sAPAP as indicated for 2 h, and afterward with TNF and ActD or cisplatin for 14 h Western blots of indicated proteins in cell lysates are shown. Numbers on the left indicate molecular weight in kDa. **C** Representative microscopy images of PMH treated as described in (**B**). Scale bar 50 µm. **D** Representative fluorescence microscopy images of propidium iodide (red) and Hoechst (blue)-stained PMH pretreated with 20 mM sAPAP, and challenged with 2 ng/ml TNF plus 30 nM ActD. Scale bar 50 µm. **E** Western Blots of mitochondrial (M) and cytosolic (C) fractions of PMH pretreated with sAPAP for 2 h and challenged with TNF plus ActD or cisplatin for 6 h. **F** Schematic illustration of APAP-mediated prevention of caspase activation after MOMP.

To investigate this intriguing phenomenon, i.e. that despite preceding apoptotic signaling caspases remain inactive, we analyzed whether APAP has a general effect on caspase activation. Thus, prior to initiating either intrinsic and extrinsic apoptosis, we treated hepatocytes for a short duration with sublethal concentrations of APAP and removed the treatment solution afterward (abbreviated as short APAP, sAPAP). sAPAP alone only caused little cell death but it enhanced TNF and Actinomycin D (ActD)-induced cell death (Fig. 2A, red line). Strikingly, although TNF-induced cell death was enhanced, sAPAP markedly and dose-dependently reduced TNF-induced caspase activity (Fig. 2A, black line). This reduction in caspase activation and associated apoptosis induction was also evidenced by Western Blots and microscopy (Figs. 2B, C, S2A). Importantly, sAPAP also prevented cisplatin-induced caspase activity while increasing cisplatin-induced cell death (Figs. 2B, C, S2B). Morphologically, dying cells following TNF/ActD or cisplatin treatment changed from apoptotic shrinkage and blebbing to necrotic swelling when pretreated with sAPAP (Fig. 2C). Inhibition of caspases by pharmacological inhibitors, such as QVD, is an established tool to promote necroptotic cell death in response to TNF and SMAC-mimetic treatment (Fig. S2C) [27]. To test whether sAPAP-induced inhibition of caspase activation similarly switches TNF-mediated apoptosis to necroptosis, we pretreated hepatocytes with sAPAP, following treatment with TNF, SMAC-mimetic BV6 and the RIPK1 and necroptosis inhibitor Nec-1 (Fig. S2D–F). Again, sAPAP prevented caspase activation (Fig. S2D) and cells switched to necrotic morphology, but the resulting cell death was not blocked by Nec-1 (Fig. S2E, F). This suggests that sAPAP shifts TNF-mediated cell death to “classical” necrosis rather than necroptosis. In summary, these data reveal that APAP actively inhibits caspase activation promoting a switch from apoptosis to necrosis. Notably, sAPAP reinforced TNF/ActD- and cisplatin-induced cytochrome c release (Fig. 2E), indicating that APAP hinders caspase activation downstream of MOMP, but independent of events that are upstream of MOMP (Fig. 2F).

### IAPs are not responsible for the lack of caspase activity after APAP treatment

**Fig. 3:**
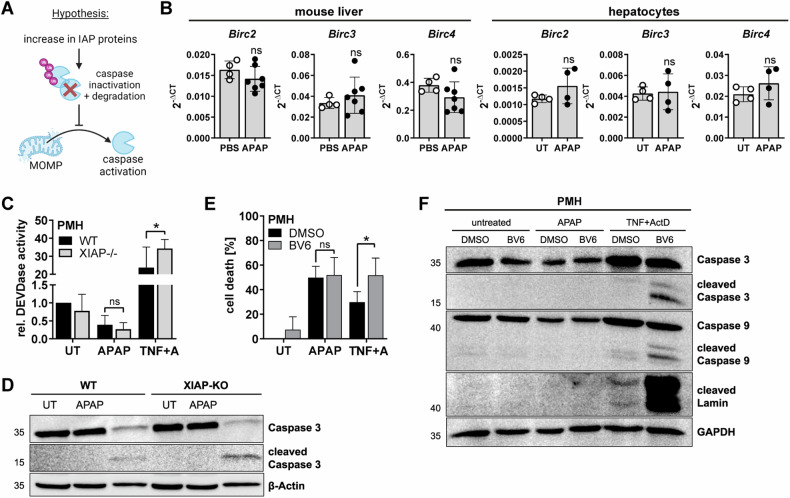
IAPs are not responsible for the lack of caspase activity after APAP treatment. **A** Schematic working hypothesis showing how IAP proteins could prevent caspase activation. **B** Transcript mRNA expression of whole liver lysates of mice treated with 500 mg/kg APAP for 6 h or of hepatocytes ex vivo treated with 20 mM APAP for 3 h. Data points display individual mice or independent replicates, *n* = 4–7. **C** DEVDase assay of cell lysates from wildtype (WT) or XIAP−/− primary murine hepatocytes (PMH) either left untreated (UT) or treated with 20 mM APAP or 2 ng/ml TNF plus 30 nM ActD for 8 h, mean + SD of *n* = 4. **D** Western Blot of cell lysates from PMH treated as described in (**C**). Numbers on the left indicate molecular weight in kDa. **E** MTT assay of PMH pretreated for 30 min with 5 µM BV6 and then treated with 20 mM APAP or 2 ng/ml TNF plus 30 nM ActD for 16 h, *n* = 5. **F** Western Blot of PMH treated as described in (**E**) for 12 h. Bar graphs display mean ± SD of independent replicates. Statistical significance was tested using unpaired Student’s *t*-test (**B**) or Two-way ANOVA with Sidak’s multiple comparison test (**C**, **E**).

Cellular Inhibitors of Apoptosis (cIAPs) are well known for their caspase-inhibiting activities [28]. Our first hypothesis how APAP could impede caspase activation was therefore that APAP may promote the upregulation of cIAPs that in turn inhibit caspase activity or force their proteasomal degradation (Fig. 3A) [29]. However, no increase in the expression of cIAP1, cIAP2, and XIAP in murine livers and hepatocytes upon APAP treatment could be detected (Fig. 3B). As XIAP can directly inhibit Caspase 9 and 3 downstream of MOMP, we next examined the sensitivity of XIAP-deficient hepatocytes to APAP, however, XIAP deficiency did also not restore caspase activity or affect cell death upon APAP treatment (Figs. 3C, D, S3). Likewise, treatment of hepatocytes with the cIAP inhibitor BV6 did not restore caspase activity in APAP-treated cells (Fig. 3E, F). In contrast, XIAP deficiency and BV6 treatment, both, massively reinforced caspase activity in TNF-treated cells. Thus, the lack of caspase activity following APAP treatment is not mediated by IAPs.

### Depletion of ATP is not responsible for the lack of caspase activity after APAP treatment

**Fig. 4:**
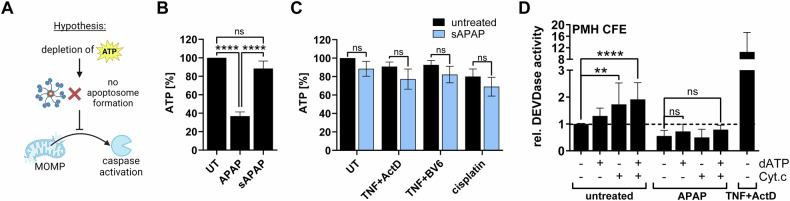
Depletion of ATP is not responsible for the lack of caspase activity after APAP treatment. **A** Schematic working hypothesis illustrating how ATP depletion could prevent caspase activation. **B** CellTiter-Glo (ATP) Assay of primary murine hepatocytes (PMH) treated for 6 h with 20 mM APAP, or for 2 h with subsequent medium change and incubation for 6 h (sAPAP), mean values + SD of *n* = 3. **C** CellTiter-Glo (ATP) Assay of PMH treated with 2 ng/ml TNF plus 30 nM ActD or 5 µM BV6, or 30 µg/ml cisplatin for 6 h, with preceding control or 20 mM sAPAP pretreatment for 2 h, mean + SD of *n* = 3. **D** DEVDase assay of cell-free cytosolic extracts (CFE) prepared from PMH left untreated or treated with 20 mM APAP or 2 ng/ml TNF plus 30 nM ActD for 6 h. CFEs were in vitro stimulated with 1 mM dATP and/or 1 mg/ml cytochrome c (Cyt.c) for 30 min prior to measurement of caspase activity, mean values + SD of *n* = 6. Bar graphs display mean ± SD with *n* as independent replicates. Statistical significance was tested using ordinary One-Way ANOVA with Tukey’s multiple comparison test (**B**) or Two-way ANOVA with Sidak’s multiple comparison test (**C**, **D**).

ATP is an important co-factor for apoptosome formation, the activation platform of Caspase 9 [25, 26]. As we previously described that APAP extensively targets mitochondrial energy production [17], we next examined whether the resulting energy crisis is responsible for the lack of caspase activity during APAP intoxication (Fig. 4A). Indeed, APAP treatment drastically reduced cellular ATP levels, but short, sublethal APAP (sAPAP) did not significantly affect ATP levels (Fig. 4B). Since sAPAP was able to prevent TNF and cisplatin-induced caspase activity (Fig. 2), this finding already indicates that a reduction in cellular ATP is likely not the underlying cause of APAP-induced caspase inhibition. The combination of sAPAP with any of the used apoptosis triggers did also not further reduce ATP levels (Fig. 4C). To more directly investigate whether reduced ATP levels are responsible for reduced caspase activation, we generated cell-free cytosolic extracts (CFEs) of untreated or APAP-treated hepatocytes and mimicked MOMP and associated caspase activation by initiating apoptosome formation with exogenous cytochrome c and dATP [30, 31]. As expected, addition of cytochrome c and dATP elevated caspase activity in CFEs of control hepatocytes (Fig. 4D). In CFEs of APAP- or TNF/ActD-treated cells, cytochrome c should be already present due to the preceding loss of mitochondrial integrity (Fig. 1E). But intriguingly, caspase activity in APAP-treated samples was even lower than in control samples, and could not be restored or significantly increased by the addition of cytochrome c and/or dATP (Fig. 4D). Hence, it is unlikely that the decline in cellular ATP following APAP treatment is responsible for the lack of caspase activity.

### Oxidative stress, but not NAPQI, inhibits caspase activation

**Fig. 5:**
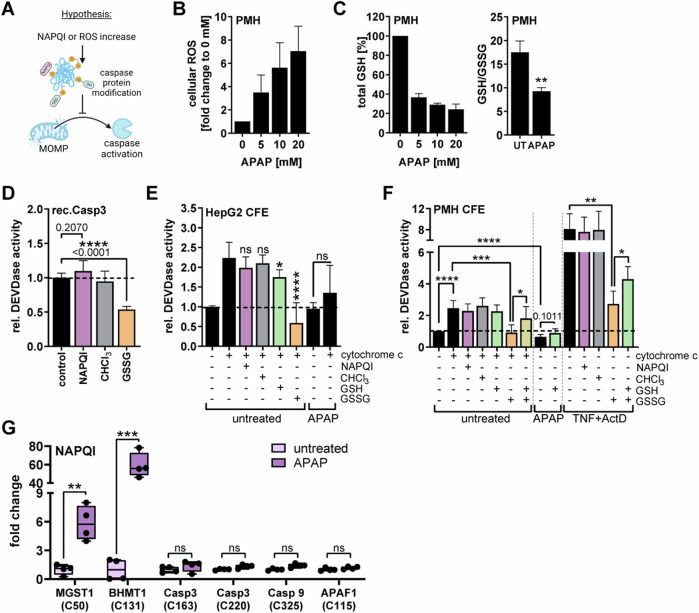
Oxidative stress, but not NAPQI, inhibits caspase activation. **A** Schematic working hypothesis illustrating that protein modifications in caspases induced by NAPQI or oxidative stress could prevent caspase activation. **B** ROS-Glo™ (cellular ROS) assay of primary murine hepatocytes (PMH) treated with APAP for 2 h, mean + SD of *n* = 4. **C** GSH/GSSG-Glo™ assay of PMH treated with indicated concentrations of APAP (left) or with 20 mM APAP (right) for 2 h, mean + SD of *n* = 3–4. **D** DEVDase Assay of recombinant Caspase 3 incubated with 25 µM NAPQI, solvent control chloroform (CHCl_3_) or 2 mM GSSG for 30 min prior to measurement, mean + SD of *n* = 4. **E**, **F** DEVDase assay of cell-free cytosolic extracts (CFE) prepared from HepG2 (**E**) or PMH (**F**) either left untreated or treated with 20 mM APAP or 2 ng/ml TNF plus 30 nM ActD for 6 h. CFEs were then in vitro stimulated with 500 µg/ml (HepG2) or 1 mg/ml cytochrome c (PMH), 25 µM NAPQI, its solvent chloroform (CHCl_3_), 2 mM GSH, or 2 mM GSSG for 30 min prior to measurement of caspase activity, mean + SD of *n* = 4–9 (for HepG2) and *n* = 5 (for PMH). **G** NAPQI cysteine modifications measured by parallel reaction monitoring (PRM) mass spectrometry from lysates of PMH treated for 4 h with 20 mM APAP, *n* = 4. Only potential oxidative cysteine modifications of the shown six selected proteins were included in the PRM search and only confidently identified and quantified modifications are shown (see methods for more details) (site of NAPQI modification is shown in brackets). Data shows normalized peak areas as fold change in respect to the respective untreated samples. Bar graphs display mean ± SD with *n* as independent replicates. Statistical significance was tested using unpaired Student’s *t*-test (**C**) or Two-way ANOVA with Sidak’s multiple comparison test (**D**, **E, F, G**).

The most fatal process during APAP toxicity is the accumulation of NAPQI and the associated drop in GSH, leading to overwhelmingly high levels of ROS [15]. NAPQI as well as oxidative stress can provoke modifications of cysteine residues in numerous target proteins. Caspase activity is critically dependent on the cysteine in its catalytically active center (Cys163 for Caspase 3, Cys277 for Caspase 9). Thus, modifications there could potentially explain the inactivation of caspases following APAP treatment (Fig. 5A). NAPQI forms covalent and irreversible protein adducts, whereas oxidative stress, i.e. an oxidizing environment, can lead to reversible and irreversible cysteine modifications. These include for example the formation of intra- or interprotein disulfide bridges, nitrosylation, and gradual oxidation, whereby S-sulfinylation and S-sulfonylation display irreversible modifications [32, 33]. The most common cysteine modification is glutathionylation, which usually serves as a detoxification mechanism of oxidized intermediates by consuming reduced GSH, but can be also caused by oxidative stress via spontaneous reaction with oxidized glutathione (GSSG). The latter reaction is termed thiol-disulfide exchange and has been only detected in conditions of low GSH and high GSSG [32, 34]. The cellular redox status is defined by the ratio of GSH and GSSG [34]. APAP treatment led to strong induction of ROS after only 2 h, and a simultaneous decline of GSH, which drastically lowered the cellular GSH/GSSG ratio (Fig. 5B, C). To test whether NAPQI or ROS affect caspase activity, we incubated recombinant Caspase 3 with NAPQI, its solvent control (chloroform), or with oxidized GSSG, and examined caspase activity. NAPQI did not affect caspase activity, but, strikingly, GSSG lowered caspase activity significantly (Fig. 5D). Also cytochrome c-mediated caspase activation in CFE of HepG2 and PMH was blocked by GSSG, but not by NAPQI (Figs. 5E, F, S4A). Similarly, also TNF/ActD-induced caspase activity was dose-dependently reduced by the administration of GSSG (Figs. 5F, S4B). Importantly, additional supplementation with reduced GSH reversed this inhibition and even increased caspase activity in CFEs from APAP-treated cells (Fig. 5F). To further exclude NAPQI adduct formation as a cause for caspase inactivity, we tested for NAPQI-modified cysteines in proteins relevant for caspase activation via targeted parallel reaction monitoring (PRM) mass spectrometry (MS). In contrast to proteins that have been previously described to be targeted by NAPQI (MGST1, BHMT1) [35, 36], Caspase 3, Caspase 9 and Apaf-1 did not show significant levels of NAPQI-modified cysteines (Fig. 5G). In summary, these data suggest that oxidative stress, rather than NAPQI adducts, prevents caspase activation following APAP treatment.

### Modulating cellular redox status enables reversible switch between necrosis and apoptosis

**Fig. 6:**
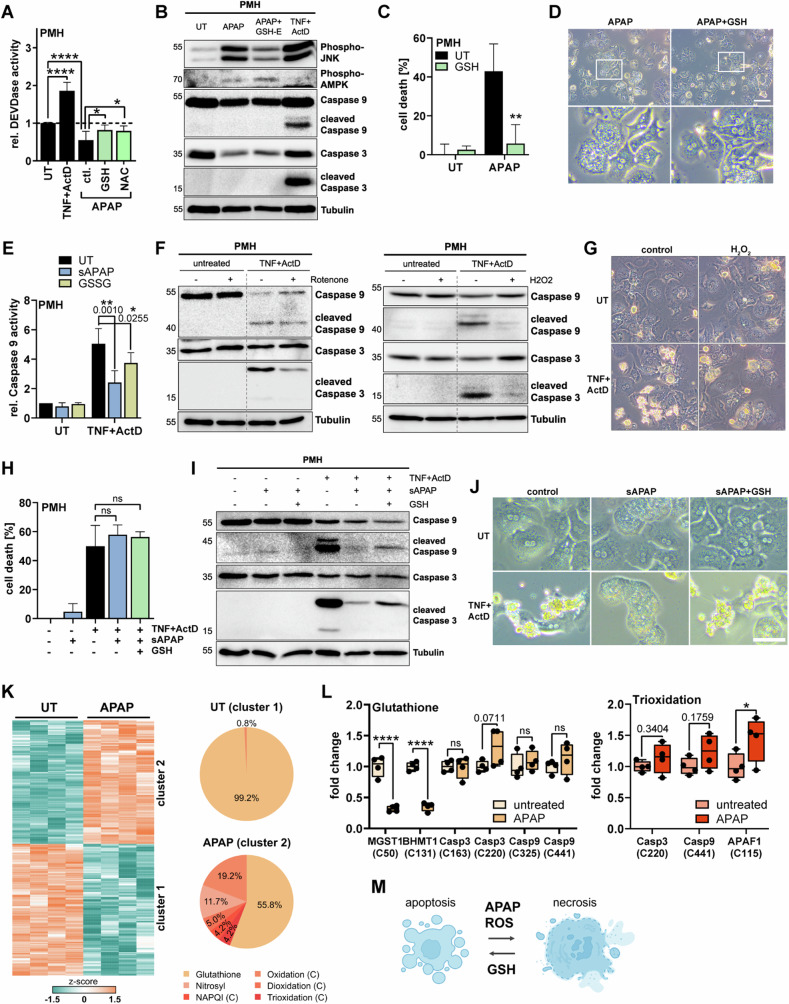
Modulating cellular redox status enables reversible switch between apoptosis and necrosis. **A** DEVDase assay of primary murine hepatocytes (PMH) left untreated (UT) or treated with either 2 ng/ml TNF plus 30 nM ActD or 20 mM APAP for 8 h with additional treatment with 5 mM glutathione ethyl ester (GSH-E, cell-permeable) or NAC after 4 h, mean + SD of *n* = 6. **B** Western Blot for indicated proteins in PMH treated as described in (**A**). **C**, **D** MTT assay (**C**) and microscopy images (**D**) of PMH treated with 10 mM APAP for 16 h (**C**) or 8 h (**D**) with additionally treatment with 5 mM GSH-E 2 h after APAP treatment, *n* = 3. Scale bar 50 µM. Inserts show magnification. **E** PMH were either pretreated with 20 mM APAP for 2 h, with subsequent removal of APAP (sAPAP), following treatment with 10 ng/ml TNF plus 30 nM ActD for 6 h, or first treatment with TNF plus ActD followed by 2 mM GSSG 2 h after TNF/ActD. Caspase 9 activity in cell lysates was measured by the Caspase 9-Glo^TM^ assay, mean values ± SD of *n* = 4. **F**, **G** Western Blots (**F**) and microscopy images (**G**) of PMH pretreated either with 1 µM Rotenone or 50 µM H_2_O_2_ for 30 min, followed by Rotenone and H_2_O_2_ removal and subsequent treatment with 10 ng/ml TNF plus 30 nM ActD for 8 h. Numbers on the left indicate molecular weight in kDa. **H** PMH pretreated with 20 mM APAP for 2 h, then APAP was removed (sAPAP) and PMH were treated with 10 ng/ml TNF plus 30 nM ActD for 8 h with additional administration of 5 mM GSH-E 2 h after TNF/ActD. Cell death was analyzed by MTT assay, mean + SD of *n* = 3. **I**, **J** Western Blot (**I**) and microscopy images (**J**) of PMH treated as described in (**H**). Scale bar 75 µm. **K** (Left) Hierarchical cluster analysis of significantly identified (*q*-value < 0.01, PTM Localization probability >0.75) and quantified (Student’s *t*-test, FDR < 0.01) oxidative cysteine modifications from lysates of PMH that were either left untreated or were treated for 4 h with 20 mM APAP using DIA-MS, *n* = 4. (Right) Venn diagrams of the significantly altered oxidative cysteine modifications identified in either the untreated samples (i.e. cluster 1 (top)) or upon APAP treatment (i.e. in cluster 2 (bottom)). **L** Relative quantification of cysteine modifications from lysates of PMH either treated for 4 h with 20 mM APAP or left untreated monitored by PRM MS, *n* = 4. Only cysteine modifications (brackets) on target proteins that were consistently identified and quantified are shown. Data is shown as relative fold change in normalized peak area relative to the untreated samples. **M** Schematic summary showing that the type of cell death can be switched from apoptosis to necrosis by treating cells with APAP or inducing ROS by other means and that cell death can be switched back to apoptosis by adding GSH. Bar graphs display mean ± SD with n as independent replicates. Statistical significance was tested using Two-way ANOVA with Sidak’s multiple comparison test (**A**, **C**, **E**, **H**, **L**).

To confirm that antioxidants can restore caspase activation upon APAP treatment in cells, hepatocytes were treated with APAP, and shortly after with a membrane-permeable GSH (GSH-E) or with the GSH precursor N-acetylcysteine (NAC). Importantly, delayed administration of antioxidants is critical to target APAP-provoked ROS/RNS and to not completely prevent NAPQI formation and oxidative stress [37]. Both antioxidants elevated caspase activity in APAP-treated cells to a level comparable to that in untreated hepatocytes (Fig. 6A, B). Of note, GSH reduced APAP-induced phosphorylation of JNK, a marker for oxidative stress, but did not affect APAP-induced phosphorylation of the energy stress sensor AMPK (Fig. 6B). NAC is a well-established antidote in the treatment of APAP-intoxicated patients as it counteracts the detrimental loss of GSH. Likewise, direct supplementation with GSH also protected cells from APAP-induced hepatocyte necrosis (Fig. 6C, D). As this also reduces the overall cellular stress, it likely also prevents initiation of apoptosis-triggering pathways and associated caspase activation (Fig. 6A). Specifically, APAP-induced oxidative stress activates JNK, which in turn leads to expression and activation of pro-apoptotic proteins [38–40], that then initiate the apoptotic signaling cascade. Indeed, GSH lowered APAP-induced BIM expression similar as JNK inhibition and diminished associated MOMP (Fig. S4D, E). Hence, as antioxidants appear to reduce the initial apoptotic signaling upon APAP, we used again our sAPAP-TNF approach comprising a more ROS-independent initial apoptotic trigger. To further examine whether excessive ROS can inactivate caspases, we replaced the short APAP pretreatment prior to apoptosis stimulation (Fig. 2) with other ROS-generating agents and tested whether they also prevent TNF-mediated caspase activation. Similar to sAPAP, also GSSG, Rotenone, H_2_O_2_, and FCCP drastically reduced Caspase 9 and Caspase 3 activation following TNF/ActD treatment (Figs. 6E, F, S4C). Alike to sAPAP, the ROS-generating agents did not protect from TNF/ActD-induced cell death but switched the mode of cell death from apoptosis to necrosis (Fig. 6G, H). Remarkably, the sAPAP-induced inhibition of caspase activity, and the associated switch from apoptosis to necrosis, could be clearly reversed by administration of the antioxidant GSH (Figs. 6H–J, S4F). Interestingly, APAP treatment thoroughly changed the pattern of cysteine modification in hepatic proteins by shifting it from mainly glutathionylation, presumably homeostatic and beneficial, to a much more diverse pattern including many ROS/RNS-related modifications (Fig. 6K). We also had a closer look at proteins relevant for caspase activation and identified Caspase 3, Caspase 9, and Apaf-1 as targets of glutathionylation and trioxidation (Fig. 6L). This suggests that oxidative stress may indeed directly impact caspase activation via cysteine modifications. In summary, we demonstrate that APAP-provoked ROS critically impedes caspase activation in response to APAP and in response to apoptotic triggers. Similar findings were made for other ROS-promoting stimuli. As this inhibition was reversed via antioxidants, we propose that the decision between apoptosis and necrosis is determined by the cellular redox homeostasis (Fig. 6M).

## Discussion

APAP overdose can result in fatal liver damage leading to acute liver failure and death [41]. A key aspect of APAP-induced liver damage is the underlying mode of cell death, i.e., oncotic necrosis. The disintegration of hepatocytes during necrosis and the release of cellular components is highly inflammatory and detrimental as it enforces rapid spreading through the tissue. Thus, APAP-induced liver damage is typically associated with immune cell activation and inflammation. Intriguingly, numerous studies have emphasized the importance of apoptotic signaling processes in response to APAP treatment [17, 21, 42–47], but there is no clear evidence that apoptosis contributes to APAP-induced hepatocyte death. This is particularly illustrated by the fact that active caspases, the hallmark of apoptosis, remain absent during APAP intoxication. Hence, we here investigated the much-discussed mode of cell death following APAP treatment and demonstrate that APAP initiates classical intrinsic apoptosis signaling, including upregulation of BH3-only proteins and induction of MOMP, but utterly fails to activate caspases. Moreover, we reveal that APAP is able to actively prevent caspase activation and even promote a switch from an initially apoptotic cell death to necrosis when administered upfront to classical apoptotic triggers. Strikingly, we identify that APAP-induced oxidative stress is the underlying reason for the observed caspase inactivation. Furthermore, our study provides evidence that particularly oxidative cysteine modifications in caspases and apoptosis-related proteins are responsible for the APAP-induced caspase inactivity. With this, we propose the idea that oxidative stress is a general decision-maker between apoptosis and necrosis.

The finding that oxidative stress dictates the mode of cell death in APAP-induced liver injury (AILI) likely applies to numerous other necrotic pathologies that involve high levels of ROS, such as ischemia-reperfusion injury, and several neurological and metabolic diseases. Moreover, our study blends in well with the concept of oxeiptosis, in which specific amounts of ROS determine the form of cell death, settling somewhere between apoptosis and necrotic forms of cell death [48, 49]. Future studies might determine a dose-response relationship between ROS levels and the mode of cell death, and examine whether this is physiologically relevant, for example in hepatic zonation. It is thus intriguing to note that many forms of drug-induced liver damage show a typical zonal progression of cell death. Furthermore, the identification on ROS as a decision-making component in hepatocyte death regulation could be pharmacologically exploited. Switching from apoptotic, immunologically ‘silent’ to more ‘violent’, necrotic, and immunostimulatory forms of cell death could improve the killing and control of solid cancers by the activation of the immune system [50]. V*ice versa*, a switch from necrotic to apoptotic cell death might be favorable to limit rapidly spreading tissue damage, as observed in AILI.

In clinical settings, apoptotic cell death is often favored over necrotic cell death due to uncontrolled consequences on tissue damage. Our data indicate that antioxidant treatment could shift APAP-induced necrosis to apoptosis, potentially mitigating liver damage in APAP-intoxicated patients. However, experimentally antioxidant treatment protected from APAP-induced cell death rather than switching from necrosis to apoptosis. Similarly, it has been shown that treatment with Mito-Tempo, a specific mitochondrial ROS scavenger, also largely protected against APAP-induced liver damage and hepatocyte necrosis, although a minor proportion of hepatocytes were dying by apoptosis [51, 52]. The difference between our reductionistic cell-based study and the in vivo experiments may largely be based in the different levels of complexity. In vivo, different cell types, including immune cells, significantly contribute to APAP-induced liver damage, and immune cell-derived factors, such as TRAIL or TNF, can themselves initiate or regulate apoptosis, making particularly kinetics of cell death very different. We thus decided to circumvent this problem by testing the effect of APAP and antioxidants on TNF-mediated apoptosis. Thereby, we could demonstrate that i) APAP prevents TNF-induced caspase activation, and that ii) antioxidants reverse this inhibition. These findings clearly contributed to mechanistically explain the paradox of APAP-induced cell death. Thus, we suggest that the translational potential of our observation lies not in the switch between cell death modalities per se, but in the strong protective effect of antioxidants during APAP intoxication. The switch from necrosis to apoptosis thus rather represents a beneficial side-effect. Until now, the antioxidant NAC is the only approved antidote for APAP-poised patients by restoring hepatic GSH levels, thereby reducing NAPQI detoxification and oxidative stress. However, NAC has several side-effects and correct dosing appears to be difficult [53]. In our experiments, we thus used primarily the GSH derivative GSH ethyl ester (GSH-E). The derivative GSH-E is more lipophilic and cell-permeable than NAC, which improves bioavailability [54, 55]. GSH-E derivatives have been furthermore shown to be more potent in reducing oxidative stress than the non-modified GSH or NAC in different oxidative stress-related diseases and conditions. Thus, GSH-E is likely also more effective than NAC or conventional GSH in preventing APAP-induced oxidative stress. Importantly, increased liposolubility of GSH-E also enables its penetration into mitochondria [56], the main source of oxidative stress upon APAP intoxication. This might represent the key advantage over NAC/GSH making GSH-E potentially similar effective as the mitochondrial ROS scavenger Mito-Tempo, previously used in in vivo studies [57]. Thus, our study emphasizes the potential of antioxidants for treating APAP-intoxicated patients and highlights the need to develop more sophisticated and directed antioxidant-based therapies, like GSH-E derivatives or Mito-Tempo.

Here, we not only demonstrate that APAP fails to promote caspase activation and apoptosis, but it also prevents caspase activation initiated by classical apoptosis triggers such as TNF or cisplatin. Our data furthermore suggests that caspases are modified and inactivated in response to APAP-induced oxidative stress. First hints that APAP impedes classical apoptosis execution and its relation to oxidative stress were already discussed in 1999 [18, 58]. The authors showed that APAP prevented Fas/CD95-mediated caspase activation and described that mitochondria-derived ROS are decisive for this inhibition [18, 58]. Intriguingly, another report recently demonstrated that oxidative stress-provoked disulfide bridges in caspase 8 caused a switch from TNF-induced apoptosis to necroptosis [59], fitting to early ideas stating that caspase activity requires a reducing environment [60, 61]. In particular, it has been shown that sufficient levels of reduced GSH are required for caspase activation [60] and that ROS-mediated caspase inhibition can be reversed by the administration of GSH [62]. Thus, our findings align well with these previous studies and additionally, finally reveal why caspases fail to be activated in APAP-intoxicated hepatocytes.

Caspases are known targets of thiol redox modifications that can compromise their activity [63]. Here, we provide good evidence that APAP treatment leads to cysteine glutathionylation and oxidation in Caspase 3, Caspase 9, and Apaf-1. First insights into caspase modifications came from studies examining nitrosylation [64, 65]. In our untargeted mass spectrometry approach we detected a substantial increase in protein nitrosylation upon APAP treatment, presumably due to accumulation of the RNS peroxynitrite [66]. However, when we specifically examined caspases and Apaf-1, we did not detect any significant nitrosylation. In addition, several studies investigated the glutathionylation of caspases as a mechanism to counteract apoptosis [67–69]. Interestingly, although Caspase 3 seems to be more glutathionylated upon APAP treatment, we found cysteine glutathionylation being overall reduced. This observed reduction in glutathionylation due to the drastic decline in GSH after APAP is also supported by earlier studies [70]. This observation also suggests that GSH-dependent glutathionylation of Caspase 3 upon APAP treatment is rather unlikely. Also, to our knowledge, glutathionylation was so far only observed in the cleaved form of Caspase 3, which does not exist in APAP-intoxicated hepatocytes. Caspase 3 might be GSSG-dependently glutathionylated (by thiol-disulfide exchange), but future studies would need to clarify this as the physiological relevance of this form of glutathionylation has been previously questioned [34]. In addition, we observed increased trioxidation (sulfonylation) in caspases and most prominently in Apaf-1. Cysteine di- and trioxidations are believed to be irreversible modifications, which would explain the difficulty in promoting caspase activation in APAP-treated hepatocytes. Thus, the addition of antioxidants to APAP-treated hepatocytes likely does not reverse these modifications but rather limits further ROS/RNS generation and additional modifications. Along these lines, it is interesting to note that the antioxidants Mito-TEMPO and NAC have a protective effect on APAP-induced liver injury, but only Mito-TEMPO was partially able to restore apoptotic cell death [51, 52]. In our own experiments, we have seen that the cell-permeable glutathione analog GSH-E almost completely prevent APAP-induced hepatocyte necrosis in vitro, yet failed to restore apoptosis, likely because a reduction in APAP-induced ROS formation also resulted in reduced upregulation of pro-apoptotic BCL-2 homologs and MOMP, and thus a trigger of caspase activation. Of note, none of the detected cysteine modifications that are significantly changed were located in the catalytically active centers of the caspases. Nonetheless, these modifications may certainly affect protein conformation and with this enzymatic activity or protein-protein interaction. All in all, our study strongly indicates that APAP treatment leads to partially irreversible oxidative cysteine modifications in caspases and Apaf-1, which critically limits their function and the associated possibility to undergo apoptotic cell death in response to APAP.

While potentially also other APAP-initiated processes could contribute to the observed inhibition of caspases, our findings support the idea that elevated expression of IAPs, drop in cellular ATP levels or accumulation of NAPQI are not responsible for the observed caspase inhibition. In this, to our knowledge, first study investigating the role of IAPs in AILI, we provide evidence that cIAP1/2 and especially XIAP are dispensable for APAP toxicity. Moreover, while in a previous study, we could circumvent the APAP-induced drop in ATP levels by several approaches, we failed to restore caspase activation in any of the conditions [17]. In addition, exogenously added dATP was also unable to restore caspase activation in cell lysates from APAP-treated hepatocytes. As NAPQI is known to target cysteines, the idea of NAPQI-caspase adducts initially seemed to be a most likely explanation for the observed inhibition of caspase activation. However, our functional experiments did not show any inhibitory effect of NAPQI on caspase activity. Furthermore, we did not detect any significant NAPQI modification in caspases or Apaf-1. In contrast, we confirmed that particularly detoxification and redox enzymes, such as glutathione-S-transferases (GST), appear to be heavily modified by NAPQI, further exacerbating APAP toxicity [8, 35, 36, 71].

In addition to controversies on the role of caspases and apoptosis in APAP-induced liver damage, a contribution of other necrotic forms of PCD during APAP intoxication, such as ferroptosis, necroptosis, and pyroptosis [20, 22, 23, 72, 73], have been discussed. While pyroptosis and necroptosis appear to be of little relevance in APAP-induced hepatocyte death [72–74], the contribution of ferroptosis is currently extensively debated [19]. During ferroptosis, pronounced oxidative stress is linked to this necrotic form of cell death via fatal lipid peroxidation in cellular membranes. Although several studies attributed ferroptosis a significant contribution to the pathology of AILI [75–79], it is now believed that minor lipid peroxidation in mitochondrial membranes represents just one of many mitochondrial perturbations that eventually result in the described cellular collapse [19]. In summary, it seems almost impossible to attribute a single predefined form of PCD to the APAP-induced cell death to due to the vast complexity of intracellular signaling processes, but exactly this complexity and the existence of critical signaling pathways justifies the term of “programmed necrosis” for APAP-induced cell death.

## Methods and materials

The detailed methods are described in the supplementary file.

## Supplementary information

Supplementary figures and methods

Supplementary Table 2

Supplementary Table 3

uncropped Western Blots

## Data Availability

The data availability is described in the supplementary file. Uncropped Western blots are found in the supplementary file.
