# Supplementary material for: Drug-induced oxidative stress actively prevents caspase activation and hepatocyte apoptosis
Source: Cell Death Dis. 2024 Sep 9;15(9):659. doi: 10.1038/s41419-024-06998-8 (PMC11381522; doi:10.1038/s41419-024-06998-8)
Supplement: Supplementary file 1 — Supplementary figures and methods [file 41419_2024_6998_MOESM1_ESM.pdf]

Supplementary Figures

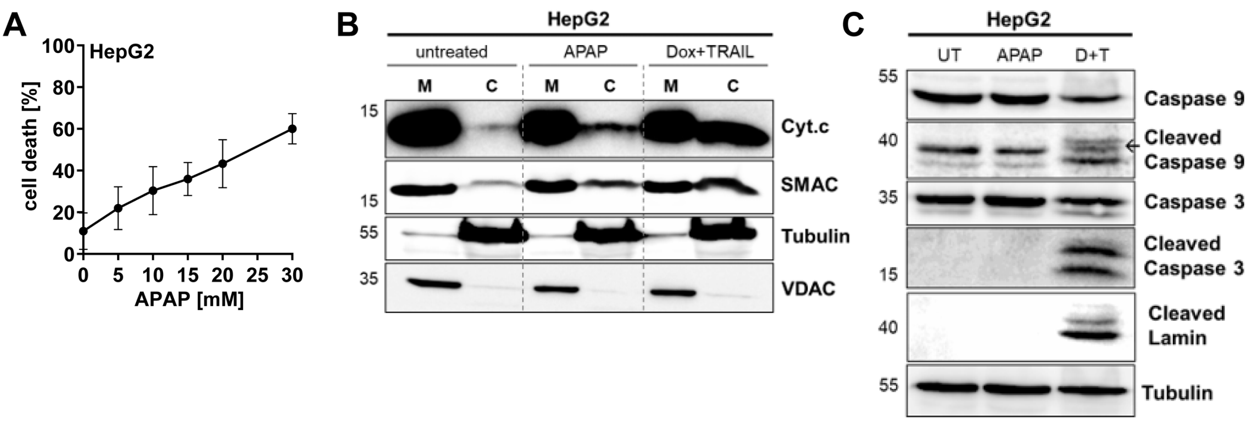

Fig. S1: APAP induces MOMP and necrotic cell death in HepG2.

A HepG2 were treated with indicated concentrations of APAP for 24h. Results of MTT assay is shown as mean ± SD of n=3 replicates.

B Western blot of mitochondrial (M) and cytosolic (C) fractions of HepG2 treated either with 20 mM APAP or 10 µg/ml Doxorubicin plus 10 ng/ml TRAIL for 6h. Numbers on the left indicate molecular weight in kDa.

C Western Blot of HepG2 left untreated (UT) or treated either with 20 mM APAP or 10 µg/ml Doxorubicin plus 10 ng/ml TRAIL for 12h.

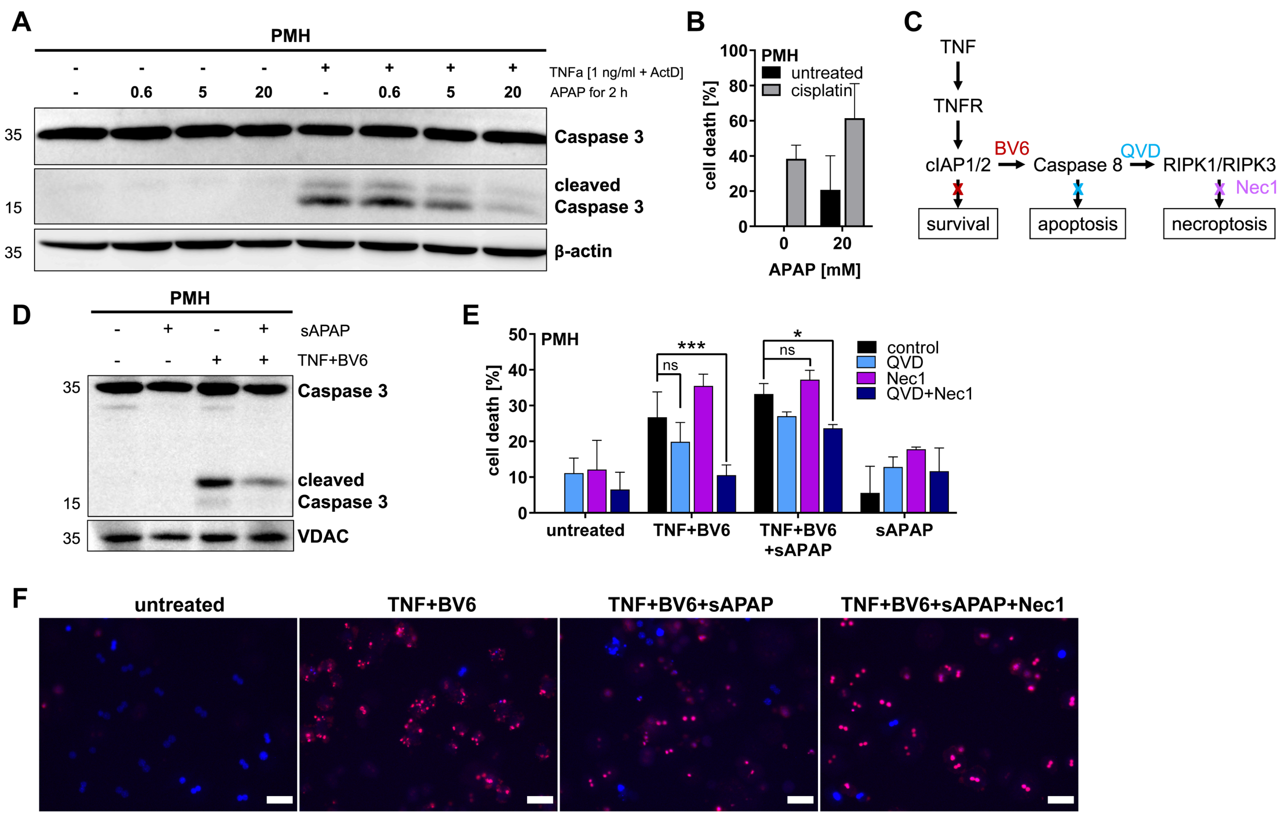

Fig. S2: APAP does not shift TNF-induced apoptosis to necroptosis but to necrosis.

A Western Blot of primary murine hepatocytes (PMH) treated for 2h with indicated concentrations of APAP, followed by removal of APAP (short APAP, sAPAP), and treatment with 1 ng/ml TNF plus 30 nM ActD for 12h. Numbers on the left indicate molecular weight in kDa

B PMH were treated for 2h with 20 mM APAP with followed by removal of APAP and treatment with 50 µg/ml cisplatin for 16h. Results of MTT assay are shown as mean + SD of n=4.

C Schematic illustration of TNF-induced survival, apoptosis, and necroptosis via treatment with specific inhibitors such as BV6, QVD and Nec1

D Western Blot of PMH treated for 2h with 20 mM APAP with subsequent removal of APAP and treatment with 50 µg/ml cisplatin for 16h.

E PMH were treated with 20 mM APAP for 2 h (sAPAP) with subsequent removal of APAP and treatment with 2 ng/ml TNF plus 5 µM BV6 for 16h, plus 30 min pre-treatment with 10 µM QVD and/or 4 µM Necrostatin-1 (Nec1). Results of MTT assay are shown as mean + SD of n=4. Statistical significance was tested using Two-way ANOVA with Sidak's multiple comparison test.

F Representative fluorescence microscopy pictures of propidium iodide (red) and Hoechst (blue)-stained PMH treated as described in (E). Scale bar 50 µm.

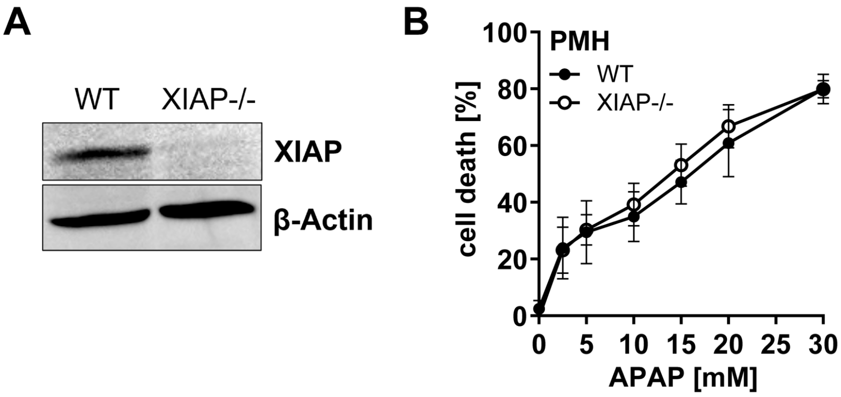

Fig. S3: XIAP deficiency does not protect hepatocytes from APAP-induced cell death.

A Western Blot of untreated primary murine hepatocytes (PMH) from wild type (WT) and XIAP-knockout mice. Numbers indicate molecular weight in kDa.

B MTT assay of WT and XIAP-/- derived PMH treated with indicated concentrations of APAP for 16h. Data points show mean ± SD of n=6 replicates.

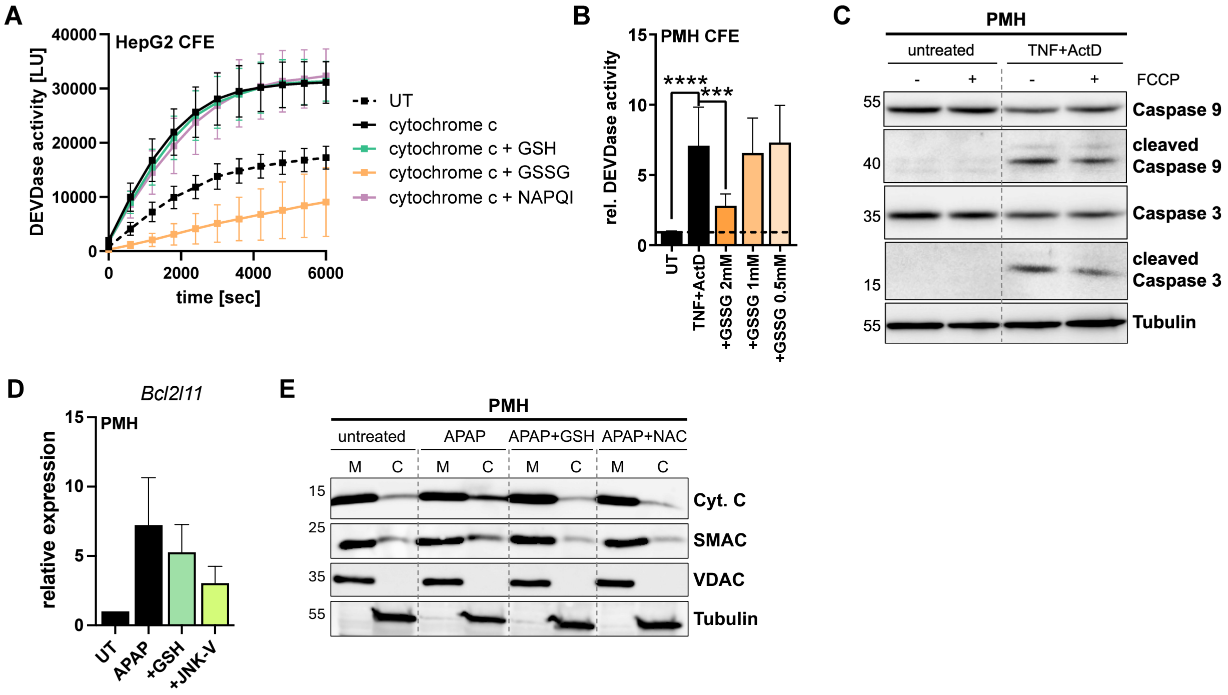

Fig. S4: Oxidative stress prevents TNF-mediated caspase activation.

A DEVDase assay cell-free cytosolic extracts (CFEs) generated from untreated HepG2 cells. CFE were then *in vitro* stimulated with 500 μg/ml, 25 μM NAPQI, 2 mM GSH, or 2 mM GSSG for 30 min prior to measurement, mean + SD of n=6.

B DEVDase assay of CFEs generated from primary murine hepatocytes (PMH) that were untreated or treated with 2 ng/ml TNF plus 30 nM ActD for 6h. The TNF-CFEs were then *in vitro* stimulated with indicated doses of GSSG for 30 min prior to measurement of caspase activity. Bar graphs display mean ± SD with n=4 replicates. Statistical significance was tested using One-way ANOVA with Tukey's multiple comparison test.

C Western Blot of PMH treated with 2 μM FCCP for 30 min, then FCCP was removed and PMH were treated with 10 ng/ml TNF plus 30 nM ActD for 8h. Numbers indicate molecular weight in kDa.

D Relative expression of Bcl2l11 (BIM gene) transcripts determined by RT-qPCR and normalized to untreated. PMH were treated for in total 6h with 20 mM APAP and 1.5h later with 5 mM GSH or 5 μM JNK-V inhibitor. Bar graphs display mean ± SD of n=3 replicates.

E Western blot of mitochondrial (M) and cytosolic (C) fractions of PMH treated for in total 6h with 20 mM APAP and 1.5h later with 5 mM GSH-E or 5 mM NAC. Numbers on the left indicate molecular weight in kDa.

Material and Methods

Animal experiments and histological analysis

Wild type C57BL/6 (WT, RRID: IMSR_JAX:000664) and XIAP-/- (B6.129P2(129S4)-Xiaptm1Hs/J, RRID: RRID:IMSR_JAX:022558) mice (male and female, 8-10 weeks old) were sourced from Jackson Laboratory and bred in-house and housed in IVC cages in the University of Konstanz's central animal facility. All procedures were ethically approved by the authorities of Baden-Württemberg, Germany under the license G20/57. In APAP experiments, mice fasted for 12 hours received 500 mg/kg i.p. injection of APAP (SIGMA) for 6 hours[1]. For TNF-induced hepatitis, mice were injected i.p. with 25 µg/kg murine recombinant TNF (Peprotec) and 1000 mg/kg D-Galactosamine (SIGMA) for 6 hours[2].

Liver lobes were fixed in 10% formalin, paraffin-embedded and sectioned in 4 µm slices. Sections were rehydrated, stained with hematoxylin and eosin (H&E), and processed as previously described[3]. For anti-cleaved Caspase 3 immunohistochemistry, rehydrated sections underwent antigen retrieval, blocking, and staining with 1:100 anti-Caspase 3 (Cell Signaling Technologies) overnight at 4 °C. Subsequent steps included incubation with 1:100 biotinylated secondary goat anti-rabbit (Jackson Immuno), ABC solution incubation (Vectastain ABC Kit, Vector laboratories), and washes in TBS-Tween. Following incubation with 1xDAB substrate solution (Roche), samples were counterstained in hematoxylin, washed, dehydrated, and mounted.

Isolation of hepatocytes

Primary mouse hepatocytes (PMH) from untreated mice were isolated by short-term storing the liver lobes in 1:75 Heparin/PBS (SIGMA) and perfusing them with 37 °C warm Buffer I (HBSS (Sigma-Aldrich), 0.1% Glucose and 2 mM EGTA) for 5-10 min, followed by warm Buffer II (HBSS (SIGMA), 5 mM $CaCl_2$ and 0.3 mg/ml Collagenase NB4 Grade (Nordmark)) for 15-20 min. The disruption of the lobe capsule by forceps released single cells into supplemented DMEM/F-12 Ham (SIGMA) media (10% FCS (SIGMA), 4 mM L-glutamine (SIGMA), 1x Pen/Strep 100x (SIGMA), 50 µg/ml gentamycin (SIGMA), 10 mg/ml Insulin Transferrin Sodium Selenite Supplement (ITS, Roche)). After filtration through 100 µM strainer and centrifugation at 75xg for 5 min, hepatocyte pellet was resuspended in 40% Percoll (GE Healthcare)/media and again centrifuged at 500xg for 10 min to remove dead cells. Pelleted, live hepatocytes were seeded in 0.3 mg/ml collagen-coated plates and cultured at 37 °C with 5% CO_2 maximum 3 days.

HepG2 cell line

Hepatoma HepG2 cells (RRID: CVCL_0027) were cultured in supplemented DMEM medium (SIGMA, 10% FCS, 2 mM L-glutamine, 20 µg/ml gentamycin) at 37 °C with 5% CO_2.

Cell death

Cell death was measured by 3-(4,5-dimethylthiazol-2-yl)-2,5-diphenyltetrazolium bromide (MTT, SIGMA) viability assay. Treated cells were incubated in 10% MTT/media solution for 1 h, then violet formazan crystals were dissolved in DMSO, and absorbance was measured at 562 nm with the Infinite® 200 PRO plate reader (TECAN). Data was normalized to untreated control.

Alternatively, cell death was assessed by propidium iodide (PI)-Hoechst co-staining. Cells were stained with 5 µg/ml PI (SIGMA) and 5 µg/ml Hoechst33342 (BioRad) for 30 min at 37 °C and visualized by fluorescence microscopy. The ratio of PI and Hoechst fluorescence per image was calculated and normalized to untreated control.

DEVDase Assay

To measure Caspase 3 activity, treated cells were washed with PBS and lysed Lysis Buffer (10 mM HEPES, 42 mM KCl, 5 mM $MgCl_2$ Hexahydrate, 0.1 mM EDTA, 0.1 mM EGTA, 0.5% CHAPS, 1 mM DTT) for 5 min on ice and for 5 min at 37 °C while shaking at 1000 rpm. Lysates were centrifuged for 10 min at 4 °C, 14000 xg, and supernatant was transferred in triplicates into black 96-well plate. Reaction Buffer (25 mM HEPES, 1 mM EDTA, 0.1% CHAPS, 3 mM DTT) including 20 µM caspase-3 substrate Ac-DEVD-AFC (Enzo Life Sciences). Fluorescence was measured as kinetic (36x10 min) at 505 nm (ex: 400 nm) with the Infinite® 200 PRO plate reader (TECAN). Activity was calculated as slope in the linear range and afterwards normalized to respective protein concentration determined by Bradford Protein Assay. Data was normalized to untreated control.

Western Blot and cytosolic and mitochondrial fractions

Treated cells or liver pieces were lysed in RIPA Buffer (50 mM Tris pH 7.4, 150 mM NaCl, 0.1% SDS, 1% NP-40, 0.5% sodium deoxycholate, fresh 1x cOmplete™ Protease Inhibitor Cocktail (Roche)). Shock-frozen murine livers were lysed by 4 Hz shaking in the TissueLyzer II (QIAGEN). Lysates were centrifuged for 15 min at 4 °C, 14000xg and total protein concentration of supernatant was determined using Pierce™ BCA Assay Kit (Thermo Fisher).

To generate cytosolic and mitochondrial fractions, cells from 10 cm dishes were lysed in cytosolic extraction buffer (1.4 mM KH_2PO_4 pH 7.2, 4.3 mM Na_2HPO_4, 250 mM sucrose, 70 mM KCl, 137 mM NaCl, 200 µg/ml digitonin, 1x cOmplete™ Protease Inhibitor Cocktail (Roche)) on ice for 5 min and supernatant (cytosolic fraction) was collected after centrifugation for 10 min at 4 °C, 1000xg. The pellet containing mitochondria was washed once and then lysed in mitochondrial lysis buffer (50 mM Tris pH 7.4, 150 mM NaCl, 2 mM EDTA, 2 mM EGTA, 0.2% Triton-X-100, 0.3% NP-40, 0.5% deoxycholic acid, 1x cOmplete™ Protease Inhibitor Cocktail (Roche)) on ice for 10 min. Mitochondrial faction was collected by

centrifugation for 10 min at 4 °C, 14000xg. Fractions were loaded in SDS-gels in a 4:1 ratio (cytosol/mitochondria).

For Western Blotting, all samples were boiled in 1x Laemmli buffer (5x: 5% SDS, 0.2 mM EDTA, 0.02% Bromophenol blue, 125 mM Tris pH 6.8, 50% Glycerol, 160 mM DTT) for 5 min at 95 °C and equal protein amounts (30-50 µg/sample) were loaded into 12% SDS-acrylamide gels (stacking gel: 5% Rotiphorese® 30 Gel (Roth), 0.13 M Tris-HCl pH 6.8, 0.1% SDS, 0.1% APS, 0.01% TEMED; separation gel: 12% Rotiphorese® 30 Gel (Roth), 0.38 M Tris-HCl pH 8.8, 0.01% SDS, 0.01% APS, 0.08% TEMED). After SDS-PAGE, proteins were wet-blotted on a PVDF membrane (Roche) in Blotting Buffer (25 mM Tris, 192 mM glycine, 0.1% SDS, 21.5% methanol), unspecific binding was blocked in 5% milk/TBS-T (137 mM, NaCl, 2.7 mM KCl, 15 mM Tris, 0.1% Tween-20) for 1 h and the membrane was incubated with primary antibodies in 5% BSA/TBS-T over night at 4 °C. After incubation with peroxidase-conjugated secondary antibodies in 5% BSA/TBS-T for 2 h, proteins were visualized with ECL solution (250 mM Luminol, 90 mM p-coumaric acid, 10 mM Tris) supplemented with 1% hydrogen peroxide in the Image Quant LAS4000 (GE Healthcare Life Sciences). A used antibodies are listed in Supplementary Table S1.

Immunoprecipitation

To study protein interaction, cells were harvested from 10 cm dishes, washed and lysed in CHAPS buffer (50 mM Tris, 150 mM NaCl, 0.5% deoxycholic acid, 1% CHAPS, 1x cOmplete™ Protease Inhibitor Cocktail (Roche), pH 8.0) on ice for 30 min. As input control, 10% of centrifuged lysate was directly boiled in 1x Laemmli buffer as described. Remaining lysate was incubated with bait or isotype antibodies for 2-3h at room temperature under constant rotation. Protein G Sepharose 4 Fast Flow beads (GE Healthcare Life Sciences) were used to immunoprecipitate bound proteins after overnight incubation at 4 °C under constant rotation. Washed beads with bound proteins were boiled in 2x Laemmli buffer at 95°C for 10 min to release bound proteins. Equal volumes were used for Western Blotting.

RT-qPCR

To measure mRNA with Real-time quantitative PCR (RT-qPCR), hepatocytes or liver pieces were lysed in TRIzol™ Reagent (Invitrogen) and RNA was isolated according to manufacturer's protocol. Remaining chromosomal DNA was removed with DNase I (NEB), and 2 µg of DNA-free RNA was reverse transcribed into cDNA by High-Capacity cDNA Reverse Transcription Kit (Applied Biosystems). RT-qPCR was performed on a StepOnePlus™ qPCR device using SYBR® Green (Applied Biosystems) and specific primers. Beta-actin CT values were used for normalization of respective samples (ΔCT(gene) = CT(gene) – CT(β-actin)). Following primers were used to detect murine mRNA: *Birc2* (cIAP1) fw GCTGACCCTACAGAGACAGTGG,

rev ATCTGCCGCTGAACCGTCTG; *Birc3* (cIAP2) fw GAATGCAGACGCAGCAATCGTG, rev GGCTGCTTTAACCACAGGCG; *Birc4* (XIAP) fw CCATGGCAGAATATGAAGCACGG, rev TCACTTTATCGCCTTCACCTAAAGC, *Actb* (β-actin) fw GATCAAGATCATTGCTCCTCCTGA, rev CAGCTCAGTAACAGTCCGCC.

ATP, GSH, and ROS Assays

Cellular ATP, GSH and ROS levels were measured with the CellTiter-Glo® 2.0 Assay, GSH/GSSG-Glo™ Assay or the ROS-Glo™ H2O2 Assay according to the manufacturer's protocols (Promega). Notably, APAP-induced cell death was not yet present at the assay end points.

Cell-free cytosolic extracts (CFE)

Cell-free cytosolic extracts (CFE) can be used to simulate MOMP *in vitro* via treatment of clean cytosolic fractions with cytochrome c. CFE were generated as previously described[4,5] with few adaptions. Briefly, cells from 15 cm dishes were harvested, washed once with cold PBS, once with cold CEB Buffer (20 mM HEPES-KOH pH 7.5, 10 mM KCl, 1.5 mM MgCl2, 1 mM EDTA, 1 mM EGTA pH 8, 10 mM DTT (fresh), 100 µM PMSF (fresh), 2 µg/ml Apronitin (fresh)) and then resuspended and allowed to swell in 500-800 µl cold CEB buffer for 15 min on ice. After swelling, cells were gently dounced 20x in a 2-ml-Dounce-type homogenizer with type-B-pestle. Lysates were centrifuged for 15 min at 4 °C, 15000xg and supernatant (=CFEs) were directly used for *in vitro* stimulation. 20 µl CFEs was incubated for 30 min at 37 °C with 5 µl stimulation mix including either 500 µg/ml (HepG2) or 1 mg/ml (PMH) bovine heart cytochrome c (SIGMA) and 10 mM dATP (SIGMA), 2 mM GSH, 2 mM GSSG, 25 µM NAPQI (SIGMA) or are fill up with CEB to 5 µl. GSH and GSSG stocks were always prepared freshly. After incubation, 20 µl/well were used for DEVDase Assay as described before.

Sample processing for mass spectrometric (MS) analysis

To analyze post-translational modifications, isolated hepatocytes were seeded in 15 cm dishes and treated on the following day with and without 20 mM APAP for 4 h before they were lysed in CHAPS Buffer (50 mM Tris, 150 mM NaCl, 0.5% deoxycholic acid, 1% CHAPS, pH 8.0) for 30 min on ice. Lysates were cleaned by centrifugation at 14 000 xg for 15 min at 4 °C. Protein concentration of cleaned lysates were between 4-6 µg/ml. Samples were prepared in four biological replicates. Proteins were precipitated by addition of 6 volumes of ice-cold acetone and incubated for 15 min at -80 °C, followed by 90 min at -20 °C. Precipitated proteins were pelleted by centrifugation at 16 000 xg for 10 min at 4 °C, and completely dried before pellets were resuspended in 8M urea. To retain reversible cysteine modifications, reduction and alkylation of cysteine residues was omitted. Urea concentration was diluted to 4M, before a Trypsin/Lys-C mix (Promega, V5071) at an enzyme-substrate ratio of 1:100 was added. Lys-C

digestion was allowed to proceed for 3 h at 37°C before samples were further diluted to 1M urea, and trypsin digestion was conducted for 18 h at 37 °C. Digestion was stopped by addition of formic acid to a final concentration of 2 % (v/v). Peptides were desalted using C18 Spin Tips (Pierce, #84850), dried by vacuum centrifugation and stored at -20 °C prior to MS analysis.

Mass spectrometric analysis – Data independent acquision

Roughly 500 ng peptides were resuspended in 5 % ACN, 0.1 % formic acid and loaded onto a QExactive HF mass spectrometer coupled with an EASY-nLC 1200 system (both Thermo Scientific) equipped with a 50 cm Acclaim PepMap C18 column (Thermo Scientific, P/N 164945). Peptides were separated across a 220 min gradient with a flow of 150 nL/min at 50 °C oven temperature. MS was operated in data independent acquision (DIA) in variable window mode using 24 windows[6]. Full mass spectra were acquired in the Orbitrap at a resolution of 120 K, an AGC target of 3e6 and 60 ms maximum injection time (max. IT) in a scan range of 300-1650 m/z. DIA scans were acquired in the Orbitrap at a resolution of 30K, an AGC target of 1e6 and max IT set to auto. Precursors were fragmented with HCD at 28 NCE.

Mass spectrometric analysis – Parallel reaction monitoring

Parallel reaction monitoring (PRM) analysis was conducted on an Orbitrap Tribrid Fusion mass spectrometer connected to an EASY-nLC1200 liquid chromatography system (both Thermo Scientific). Peptides were separated across an 80 min gradient at 300 nL/min on a 15 cm Acclaim PepMap C18 column (Thermo Scientific, P/N 164943). Full mass spectra were acquired in the Orbitrap at 120 K resolution with an AGC target of 4e5 and 60 ms max. IT in a scan range of 295-1450 m/z. Precursors were isolated in the quadrupole with an isolation width of 1.6 m/z and fragmented with HCD at 28 NCE. PRM scans were acquired in the Iontrap in rapid scan mode with a defined scan range of 150-1250 m/z, an AGC target of 1.5e4 and max. IT set to auto[7]. Loop control was set to all. The Isolation list of all precursors of interest was prepared in Skyline and set to include all peptides of the indicated proteins (P70677, Q8C3Q9, O88879, Q91VS7, O35490, Q9D172, P62897) that contained a cysteine with the defined modifications (NAPQI, Glutathione, trioxidation, nitrosylation). To minimise cycle time, this list was split into four separate lists which were used for four consecutive measurements.

Data analysis – Data obtained in DIA mode

Data acquired by DIA was analysed in Spectronaut (v. 18.6.231227.55695) in directDIA mode. BGS Factory settings were used with minimum peptide length set to 6 amino acids and variable modifications of cysteine residues to include oxidation, dixodation, trioxidation, Glutathione, Nitrosyl and NAPQI. Spectra were searched against the Uniprot database of Mus musculus (downloaded 2023/11/17). In the quantification tab, normalization filters to keep all cysteine

modifications were included and the PTM workflow with a probability cut-off of 0.75 was enabled. Results were exported as PTM pivot report and further statistical analysis was performed with Perseus[8]. Only peptides modified at cysteines that were identified in 3 of 4 replicates were kept for further analysis. A two-sample t-test (S0 = 0, FDR < 0.01) was performed and only significantly altered PTMs were retained, z-score normalized and submitted to hierarchical cluster analysis.

Data analysis – Data obtained in PRM mode

PRM data was analysed using Skyline (version 23.1.0.38). Only peptides with no missed cleavages were included, ion match tolerance was set to 0.4 m/z and all MS levels were used for quantification. Only identifications with mass accuracy ≤ 10 ppm and idotp >0.8 were considered for quantification. Peak areas were normalized to equalized medians.

Data availability

The MS data (raw files, Skyline and Spectronaut search output files) have been deposited to the ProteomeXchange Consortium via the PRIDE partner repository9[9] with the dataset identifier PRIDE: PXD050155 (Username: reviewer_pxd050155@ebi.ac.uk, Password: ZewSM2c2).

Statistics and data display

If not stated otherwise, data are means ± SD of minimum 3 independent experiments with each minimum technical duplicates. GraphPad Prism 8 software was used for statistical analysis and data visualization. Statistical significance was calculated by unpaired two-tailed Student's test when comparing two groups, one-way or two-way ANOVA when comparing three or more dependent groups with suitable post-hoc tests (Tukey's or Sidak's). Statistical significance is displayed as p-values with ≤ 0.05 being statistically significant (* = p<0.05, ** = p<0.01, *** = p<0.001, **** = p<0.0001).

References in Supplements

1 Mossanen JC, Tacke F. Acetaminophen-induced acute liver injury in mice. *Lab Anim* 2015; **49**: 30–6.

2 Hamesch k., Borkham-Kamphorst E, Strnad P, Weiskirchen R. Lipopolysaccharide-induced inflammatory liver injury in mice. *Lab Anim* 2015; **49**: 37–46.

3 Lambrecht R, Rudolf F, Ückert AK, Sladky VC, Phan TS, Jansen J *et al.* Non-canonical BIM-regulated energy metabolism determines drug-induced liver necrosis. *Cell Death Differ* 2023. doi:10.1038/s41418-023-01245-7.

4 Martin SJ, Newmeyer DD, Mathias S, Farschon DM, Wang HG, Reed JC *et al.* Cell-free reconstitution of Fas-, UV radiation- and ceramide-induced apoptosis. *EMBO J* 1995; **14**: 5191–5200.

5 Slee EA, Harte MT, Kluck RM, Wolf BB, Casiano CA, Newmeyer DD *et al.* Ordering the cytochrome c-initiated caspase cascade: hierarchical activation of caspases-2, -3, -6, -7, -8, and -10 in a caspase-9-dependent manner. *J Cell Biol* 1999; **144**: 281–92.

6 Bruderer R, Bernhardt OM, Gandhi T, Xuan Y, Sondermann J, Schmidt M *et al.* Optimization of Experimental Parameters in Data-Independent Mass Spectrometry Significantly Increases Depth and Reproducibility of Results. *Mol Cell Proteomics* 2017; **16**: 2296–2309.

7 Heil LR, Remes PM, MacCoss MJ. Comparison of Unit Resolution Versus High-Resolution Accurate Mass for Parallel Reaction Monitoring. *J Proteome Res* 2021; **20**: 4435–4442.

8 Tyanova S, Temu T, Sinitcyn P, Carlson A, Hein MY, Geiger T *et al.* The Perseus computational platform for comprehensive analysis of (prote)omics data. *Nat Methods* 2016; **13**: 731–40.

9 Perez-Riverol Y, Bai J, Bandla C, García-Seisdedos D, Hewapathirana S, Kamatchinathan S *et al.* The PRIDE database resources in 2022: a hub for mass spectrometry-based proteomics evidences. *Nucleic Acids Res* 2022; **50**: D543–D552.

Supplementary Tables

Table S1 Primary and secondary antibodies for Western Blot

Antibodies	Source	Identifier
Rabbit polyclonal anti-Caspase 3	Cell Signaling Technology	Cat: #9662; RRID: AB_331439
Mouse monoclonal anti-Caspase 9 (5B4)	Enzo Life Sciences	Cat: #ADI-AAM-139; RRID: AB_10614959
Rabbit polyclonal anti-cleaved Caspase 3 (Asp175)	Cell Signaling Technology	Cat: #9661; RRID: AB_2341188
Rabbit polyclonal anti-cleaved Lamin A	Cell Signaling Technology	Cat: #2035; RRID: AB_2234647
Rabbit monoclonal anti-BIM (C34C5)	Cell Signaling Technology	Cat: #2933; RRID: AB_1030947
Mouse monoclonal anti-Cytochrome c (7H8.2C12)	BD Pharmigen™	Cat: #556433; RRID: AB_396417
Rabbit monoclonal anti-phospho-JNK (Thr183/Tyr185) (81E11)	Cell Signaling Technology	Cat: #4668; RRID: AB_823588
Rabbit polyclonal anti-SMAC/Diablo	SIGMA	Cat: #S0941; RRID: AB_477487
Rabbit polyclonal anti-VDAC	Cell Signaling Technology	Cat: #4866; RRID: AB_2272627
Mouse monoclonal anti-Noxa (114C307.1)	Enzo Life Sciences	Cat: # ALX-804-408-C100; RRID: AB_2052079
Rabbit monoclonal anti-phospho-AMPK (Thr172) (40H9)	Cell Signaling Technology	Cat: #2535; RRID: AB_331250
Rabbit polyclonal anti-Bax	Cell Signaling Technology	Cat: #2772; RRID: AB_10695870
Mouse monoclonal anti-BAX (6A7)	Santa Cruz Biotechnology	Cat: # sc-23959; RRID: AB_626728
Rabbit monoclonal anti-Apaf-1 (D5C3)	Cell Signaling Technology	Cat: #8969; RRID: AB_10859039
Mouse monoclonal anti-α-Tubulin (B-5-1-2)	SIGMA	Cat: #T5168; RRID: AB_477579
Mouse monoclonal anti-β-actin	SIGMA	Cat: #A5441; RRID: AB_476744
Rabbit polyclonal anti-GAPDH (FL-335)	Santa Cruz Biotechnology	Cat: # sc-25778; RRID: AB_10167668
Peroxidase AffiniPure Goat Anti-Mouse IgG	Jackson Immuno Research	Cat: #115-035-174; RRID: AB_2338512
Peroxidase AffiniPure Goat Anti-Rabbit IgG	Jackson Immuno Research	Cat: #111-035-144; RRID: AB_2307391
