# Supplementary material for: Drug-induced oxidative stress actively prevents caspase activation and hepatocyte apoptosis
Source: Cell Death Dis. 2024 Sep 9;15(9):659. doi: 10.1038/s41419-024-06998-8 (PMC11381522; doi:10.1038/s41419-024-06998-8)

Fig. 1C

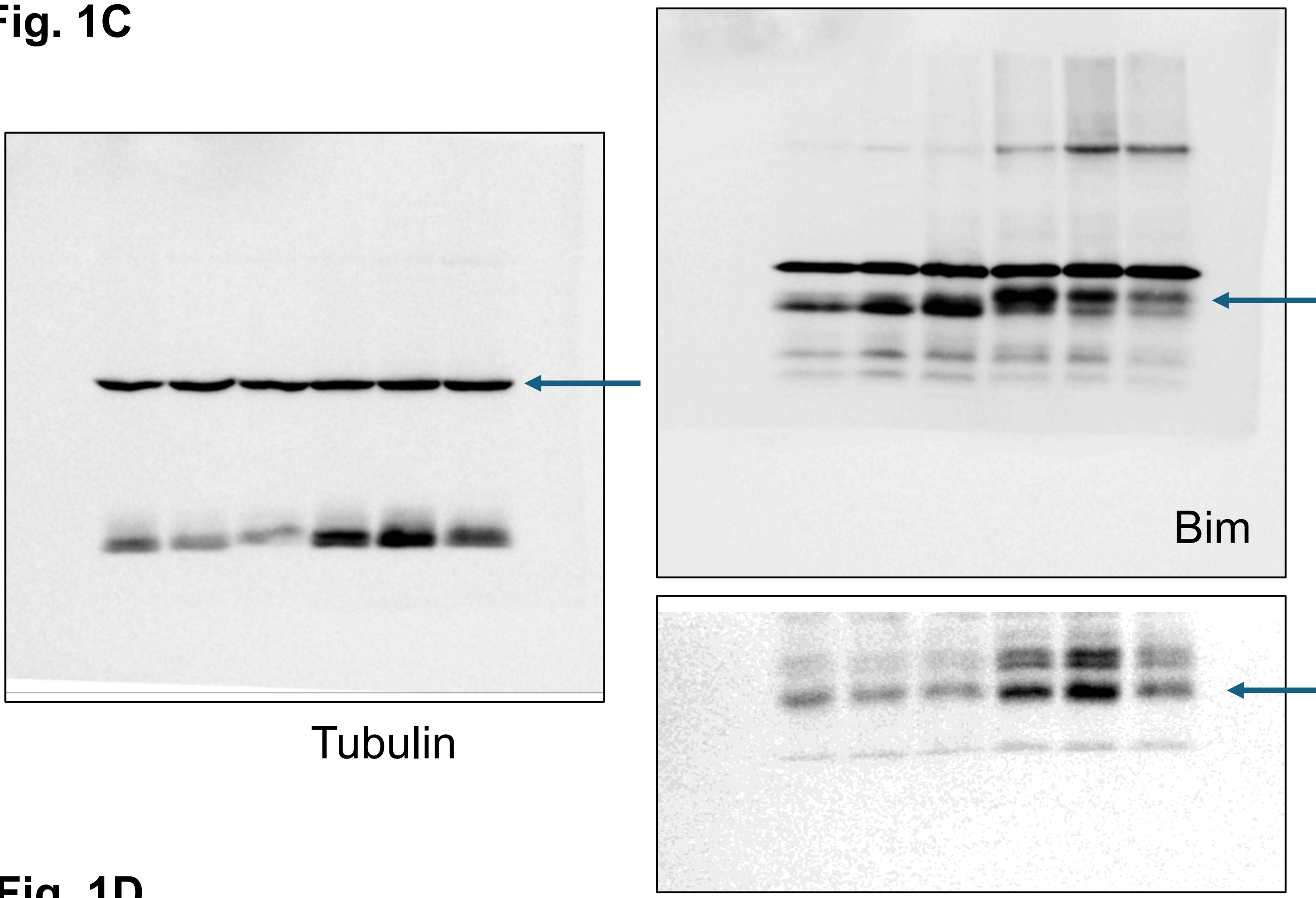

Noxa

Fig. 1D

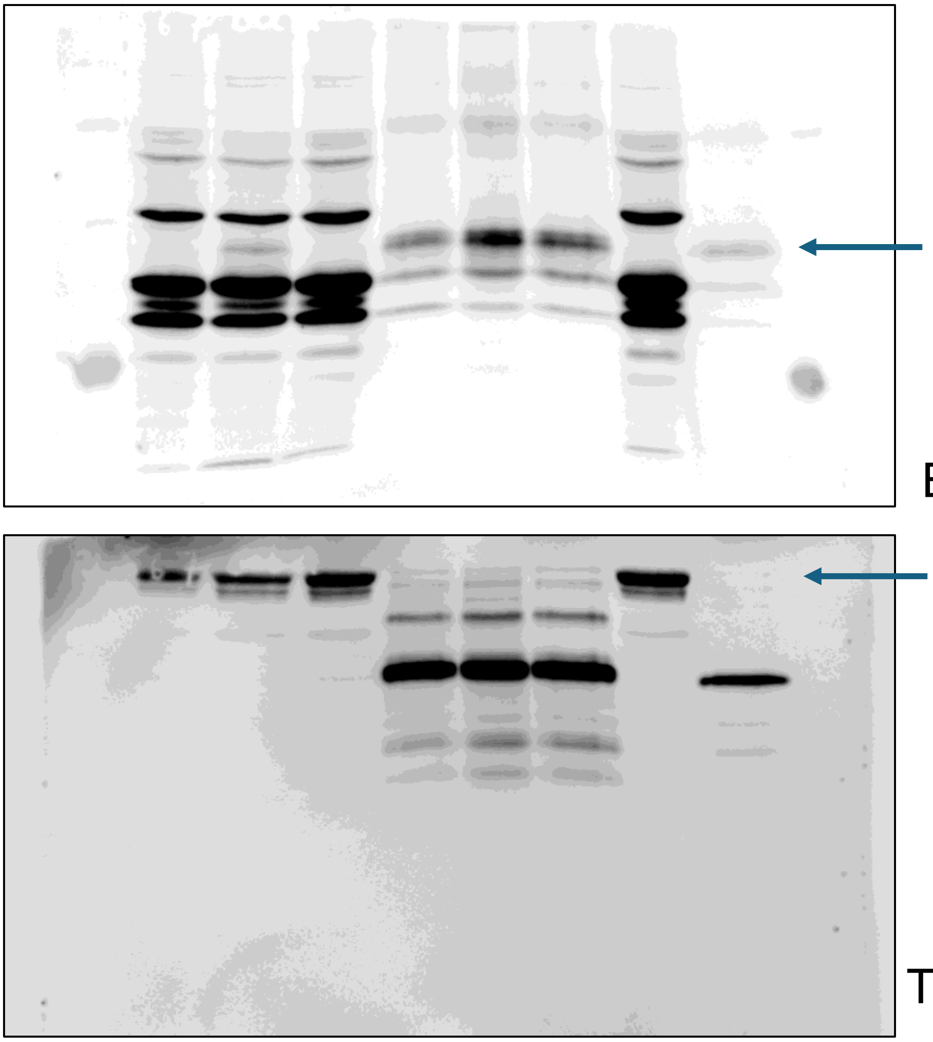

Bax

Tubulin

Fig. 1E

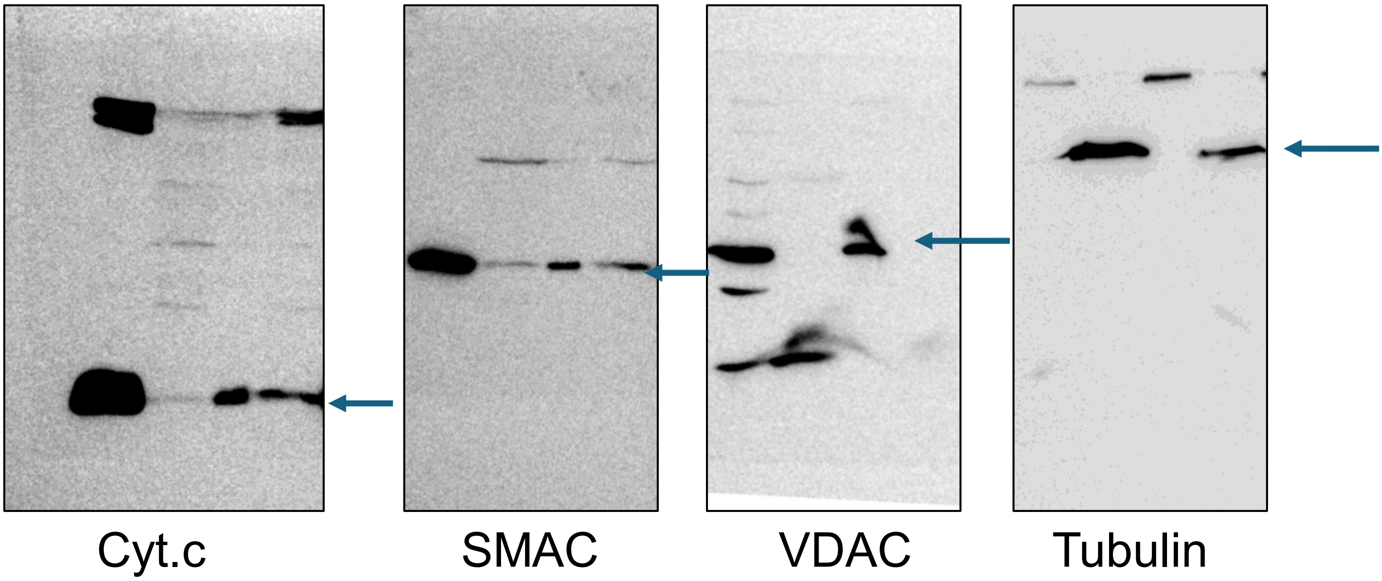

Fig. 1F

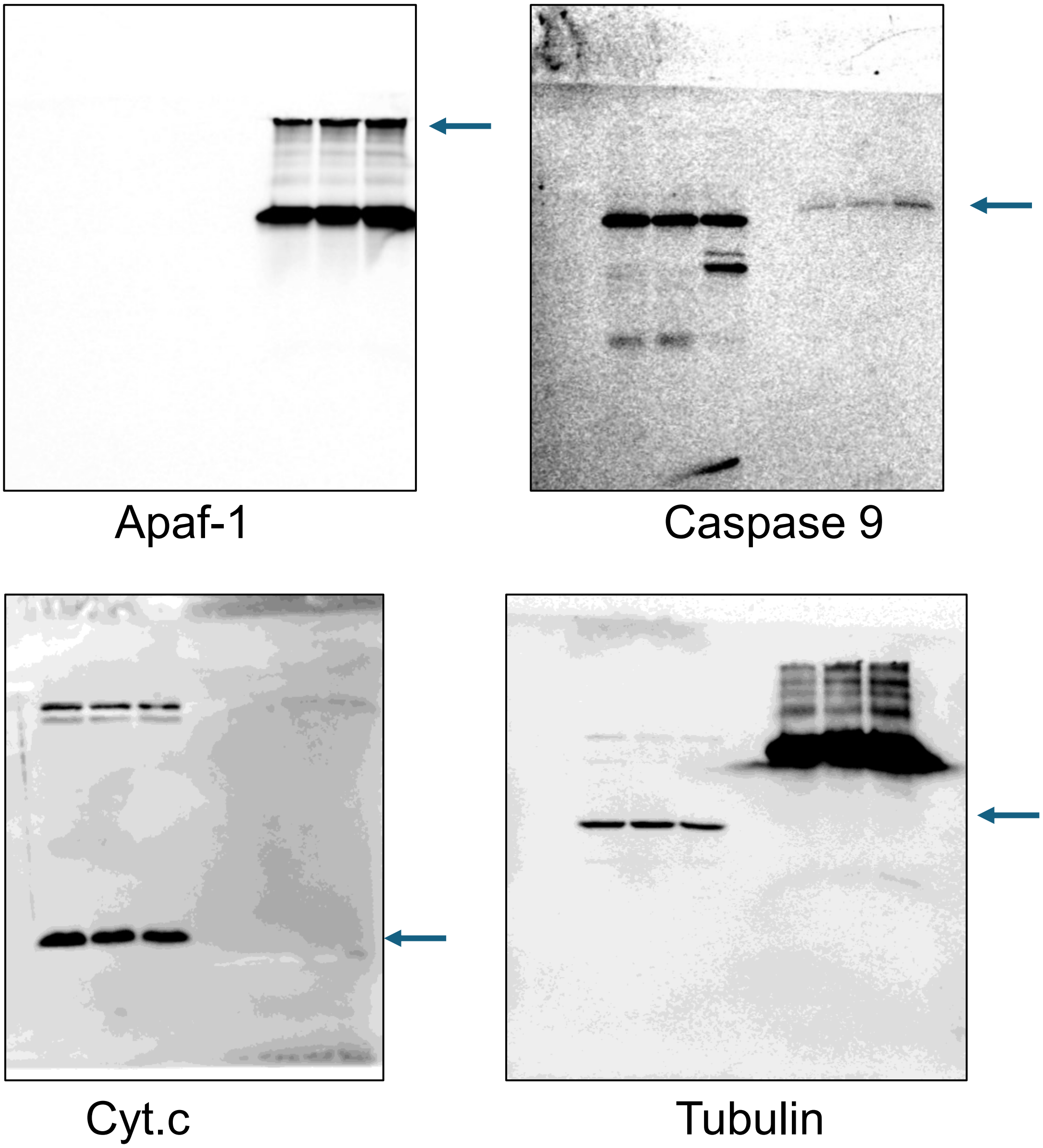

Fig. 1G

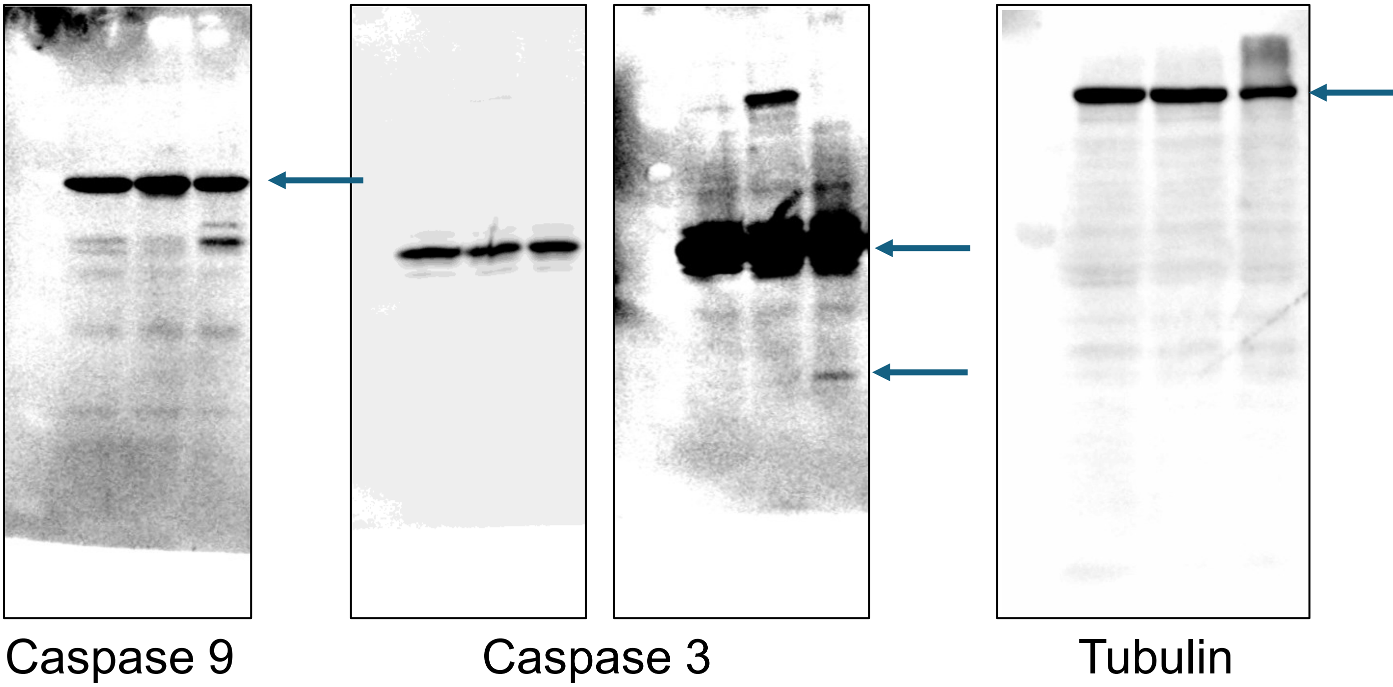

Fig. 2B TNF+ActD

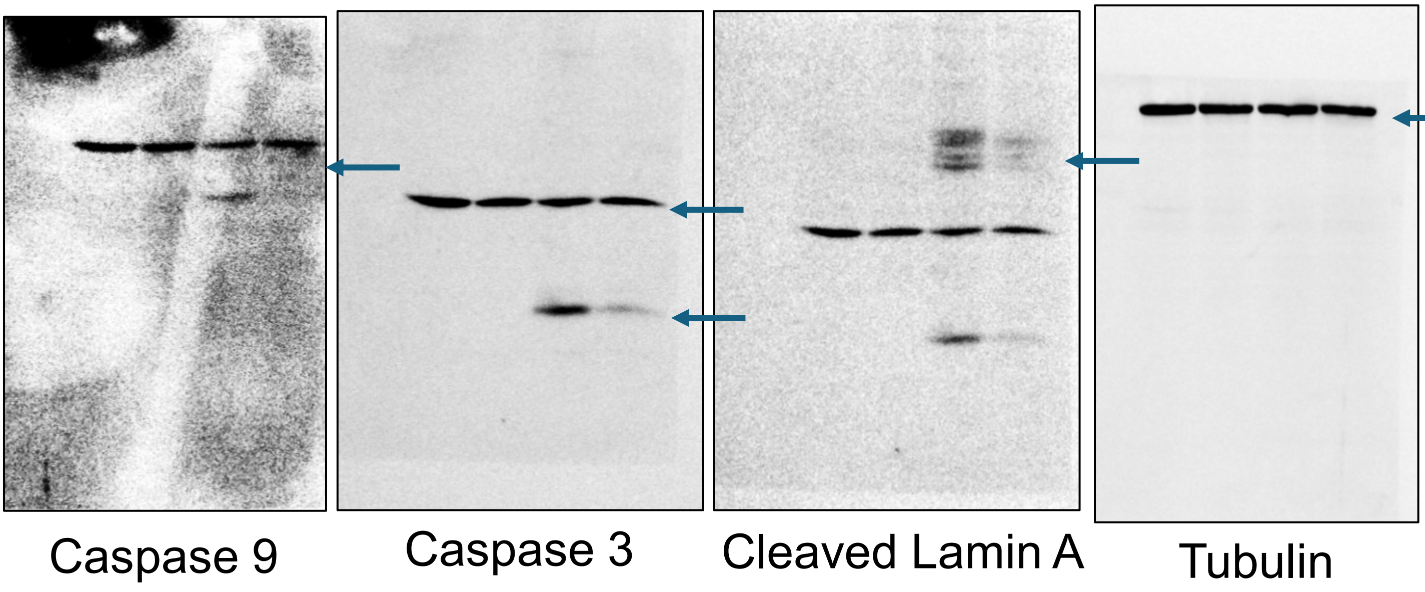

Fig. 2B cisplatin

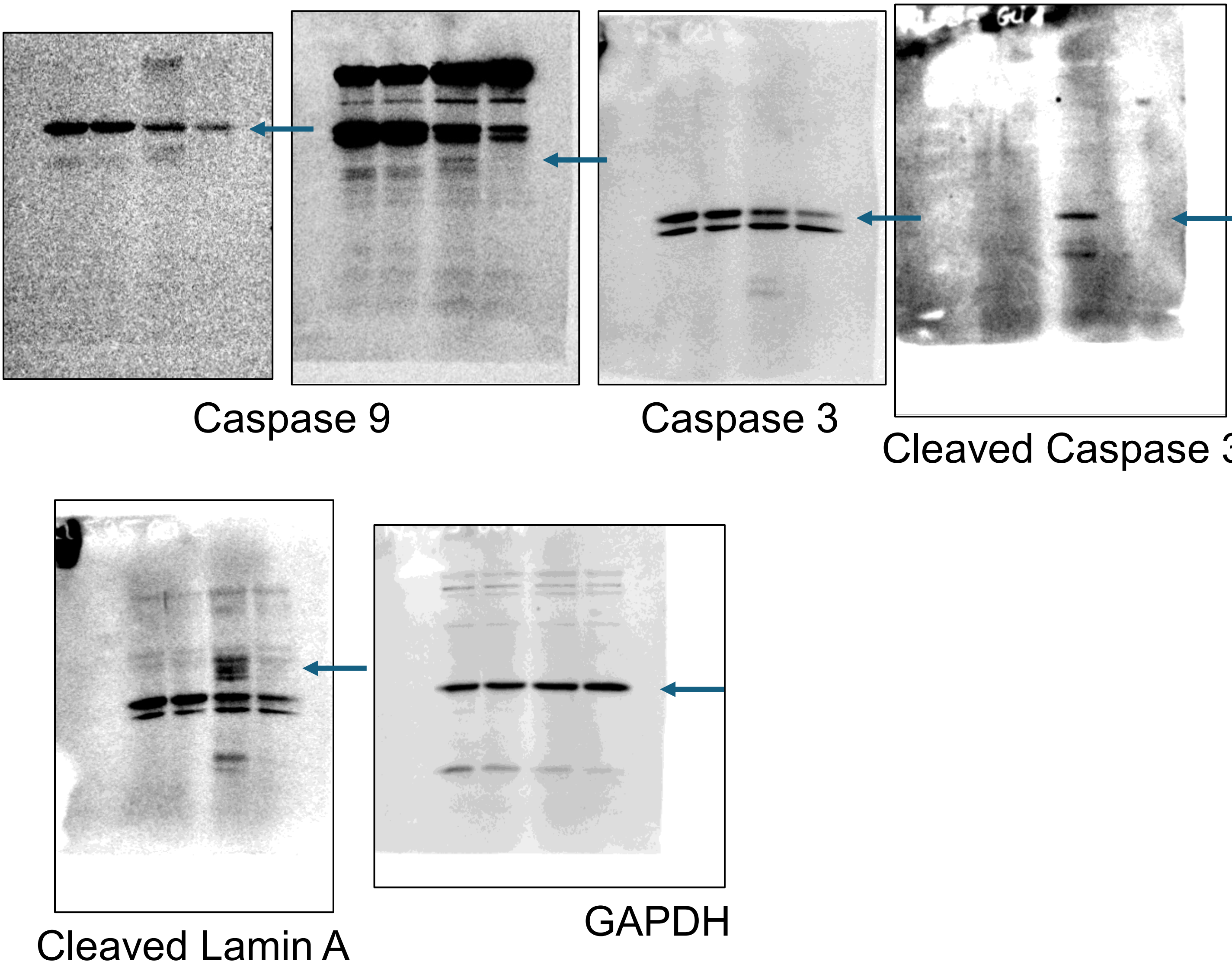

Fig. 2E TNF+ActD

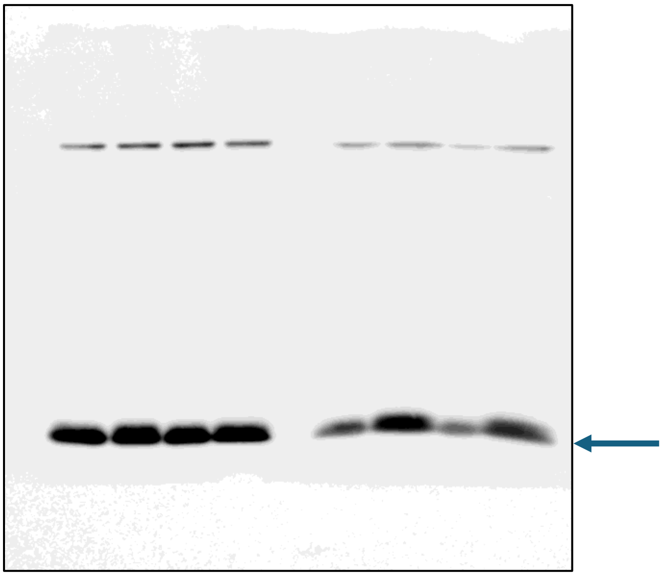

Cytc

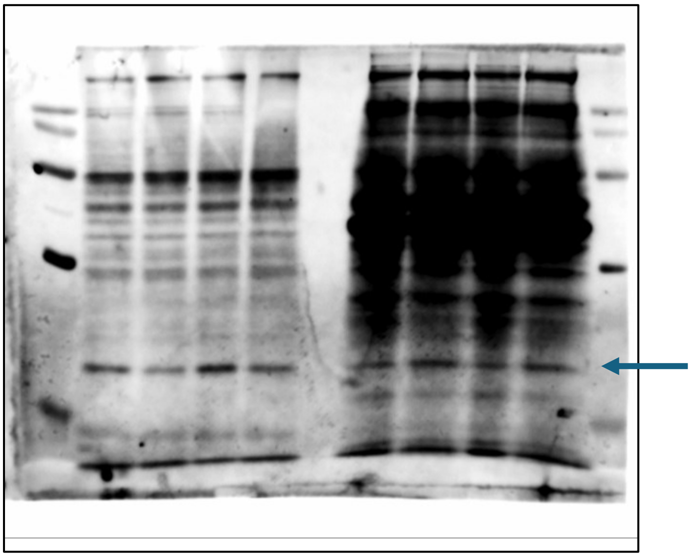

SMAC

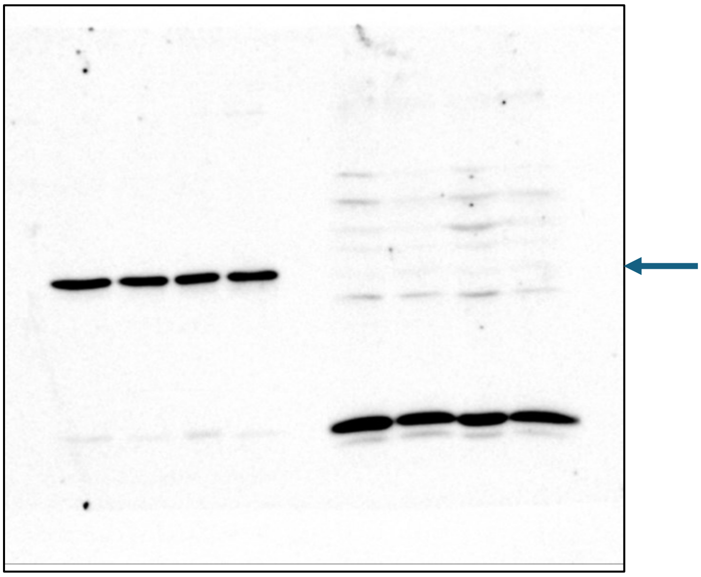

VDAC

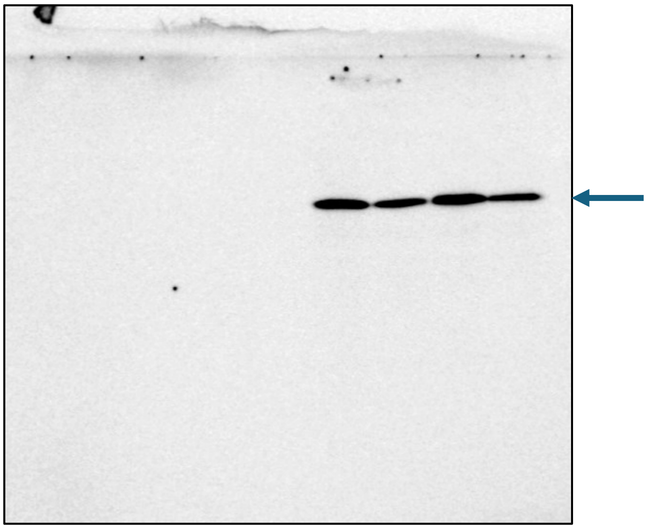

Tubulin

Fig. 2E cisplatin

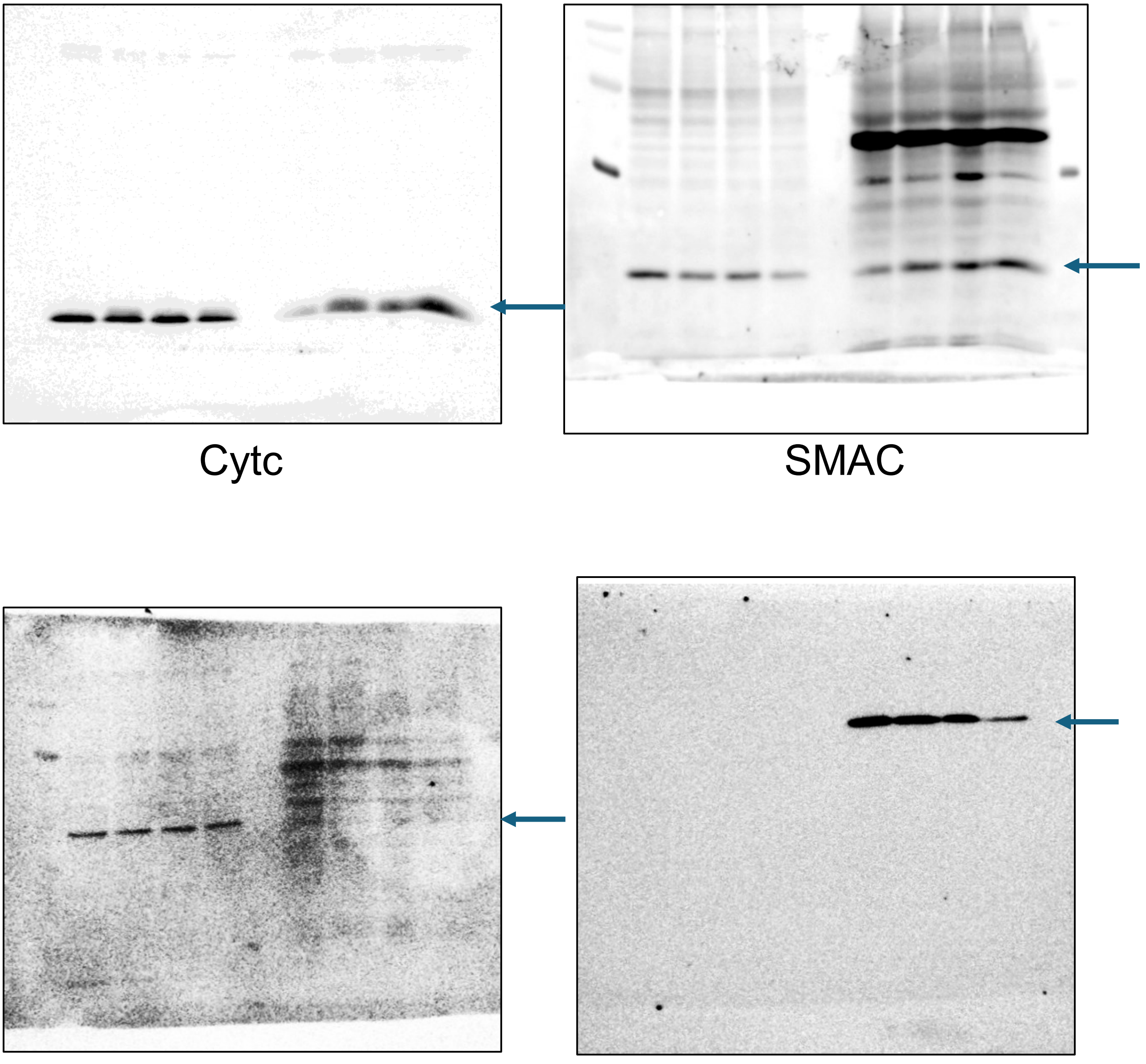

Cytc SMAC

VDAC Tubulin

Fig. 3D

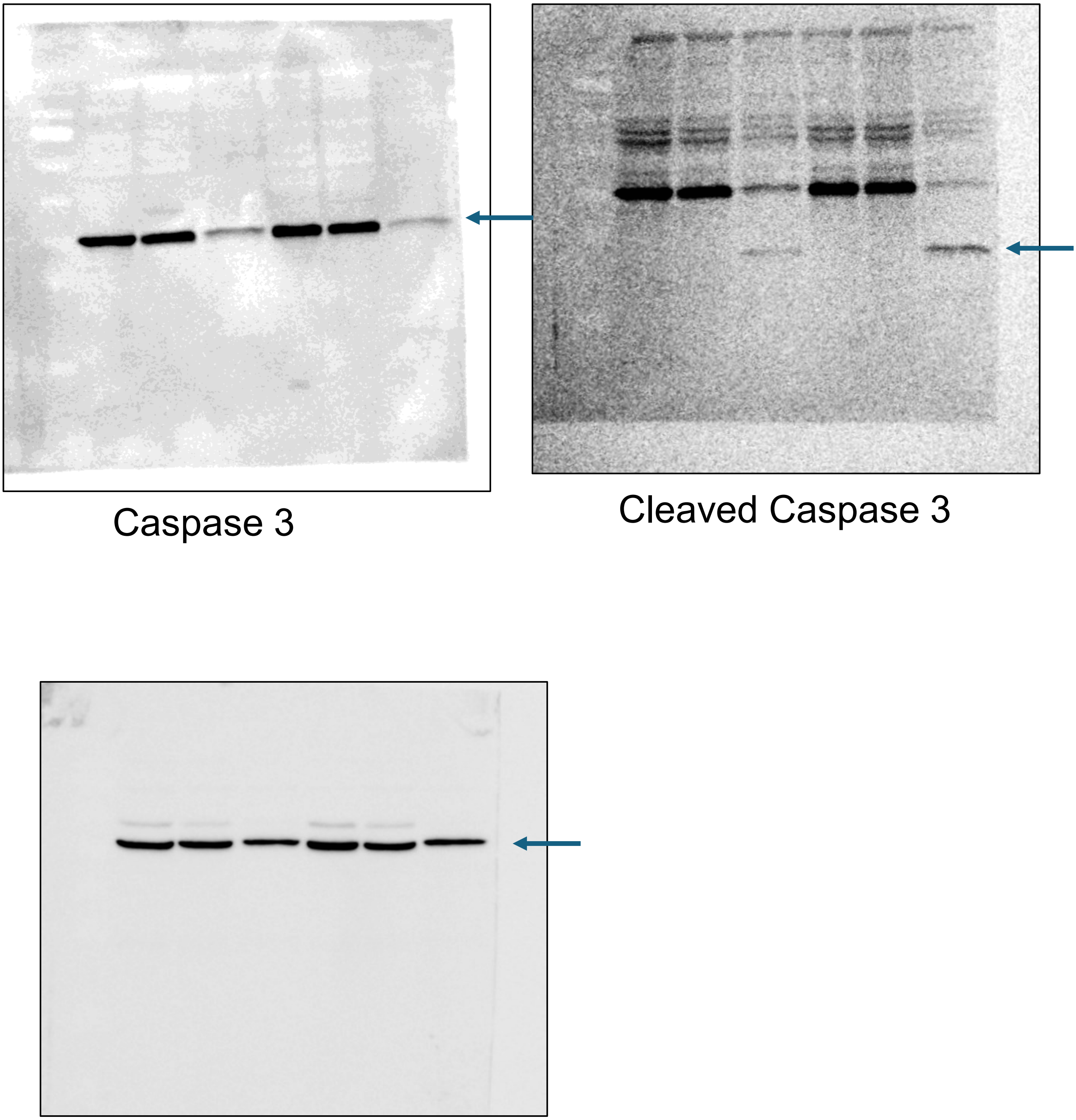

Caspase 3

Cleaved Caspase 3

B-actin

Fig. 3F

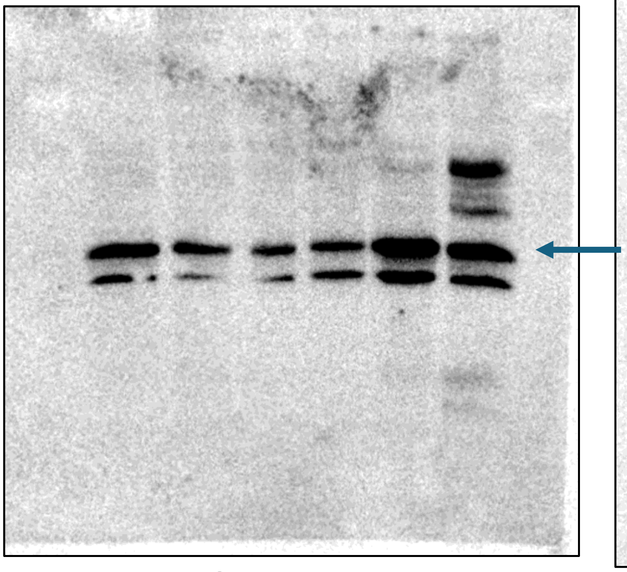

Caspase 3

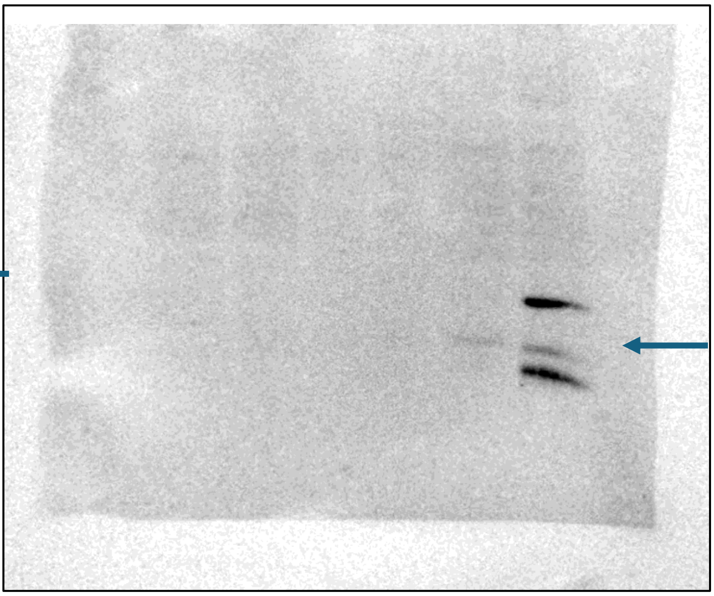

Cleaved Caspase 3

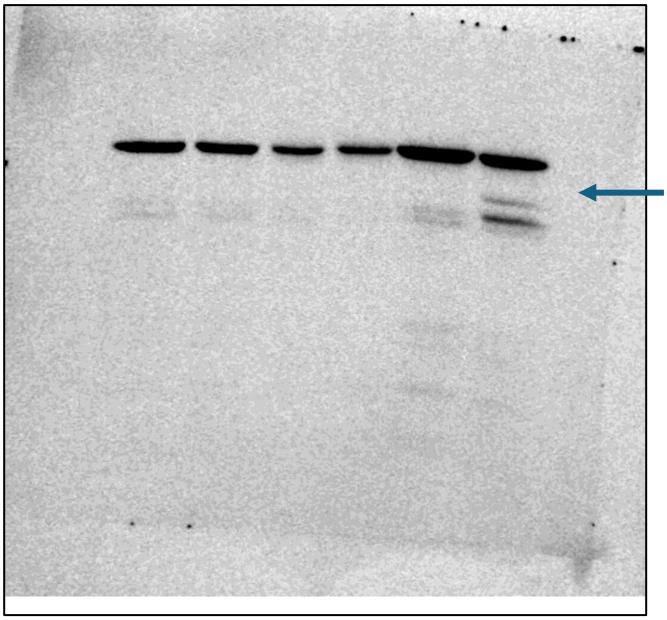

Caspase 9

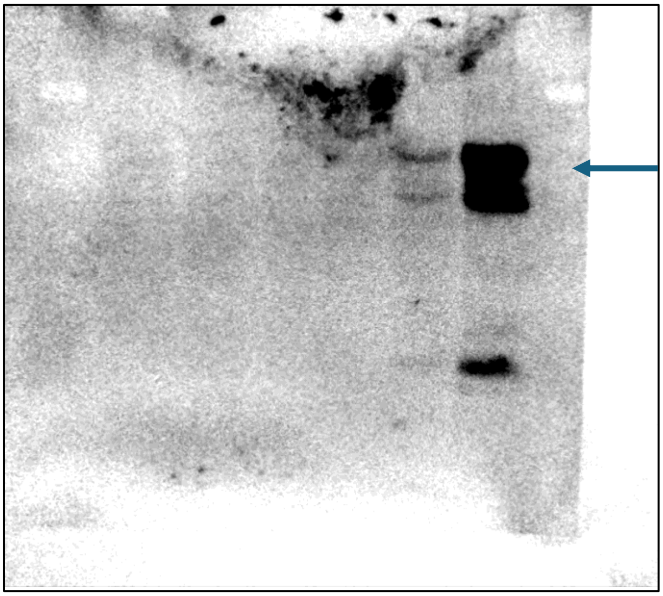

Cleaved
Lamin A

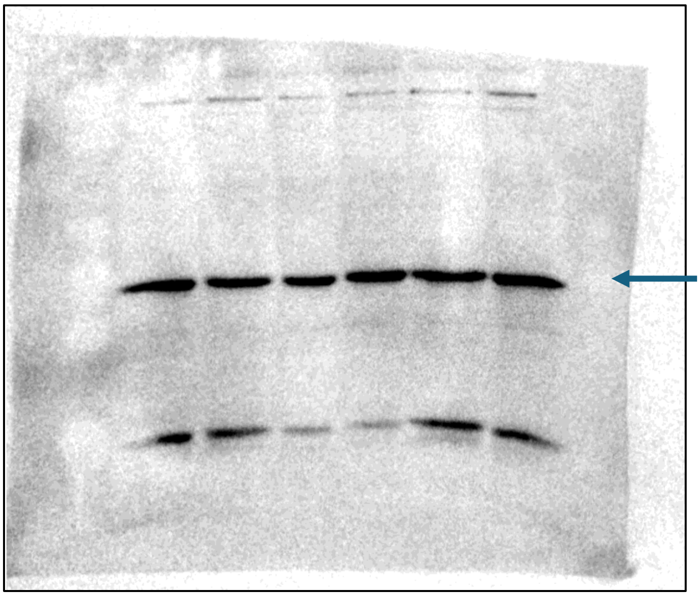

GAPDH

Fig. 6B

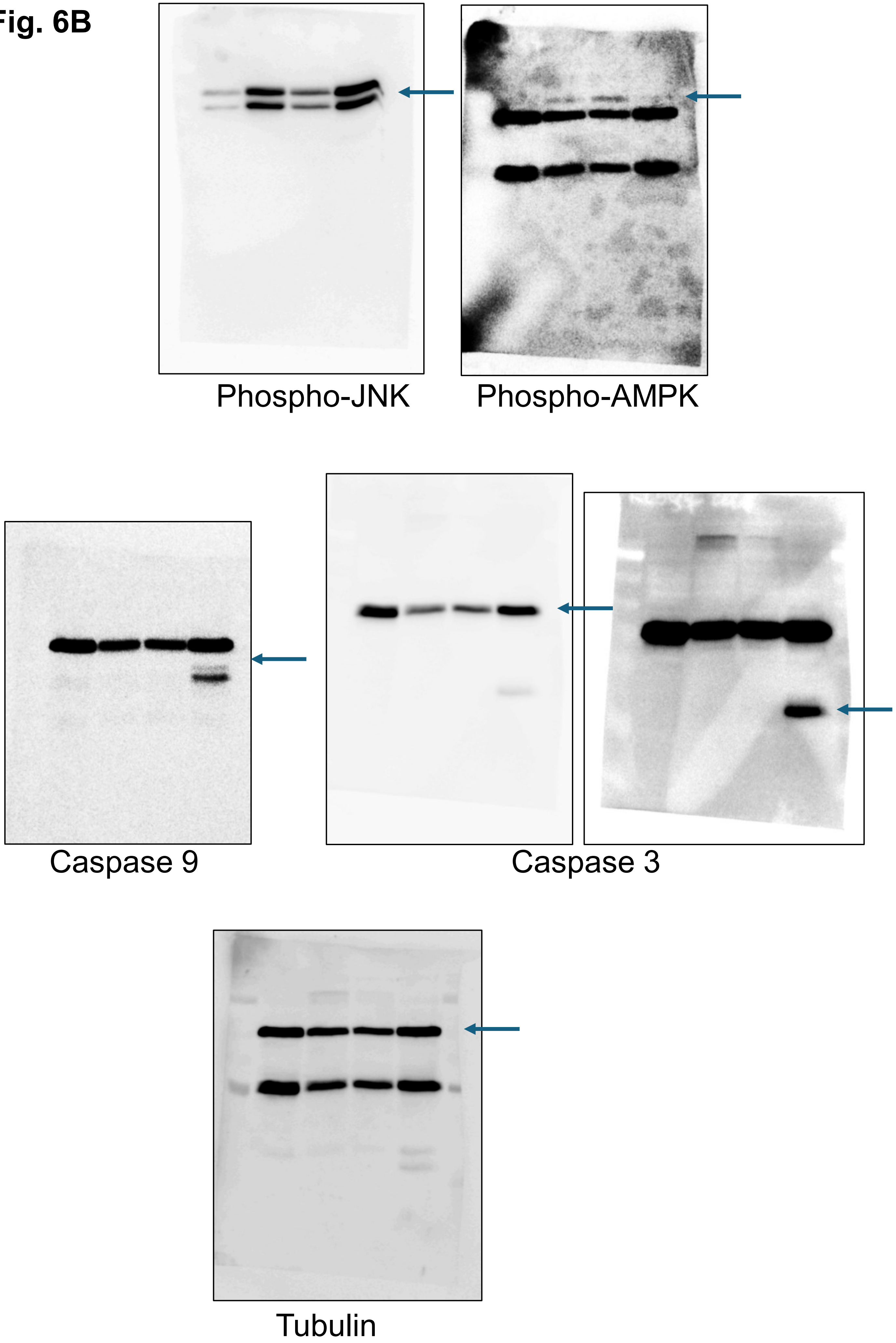

Fig. 6F Rotenone

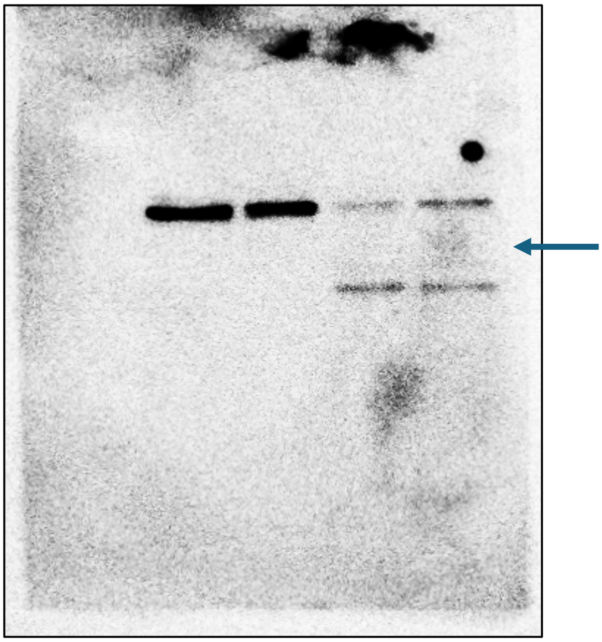

Caspase 9

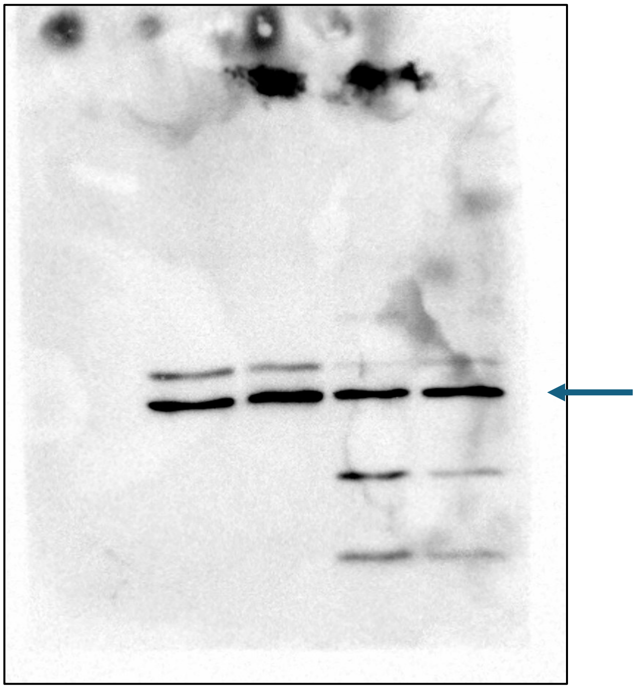

Caspase 3

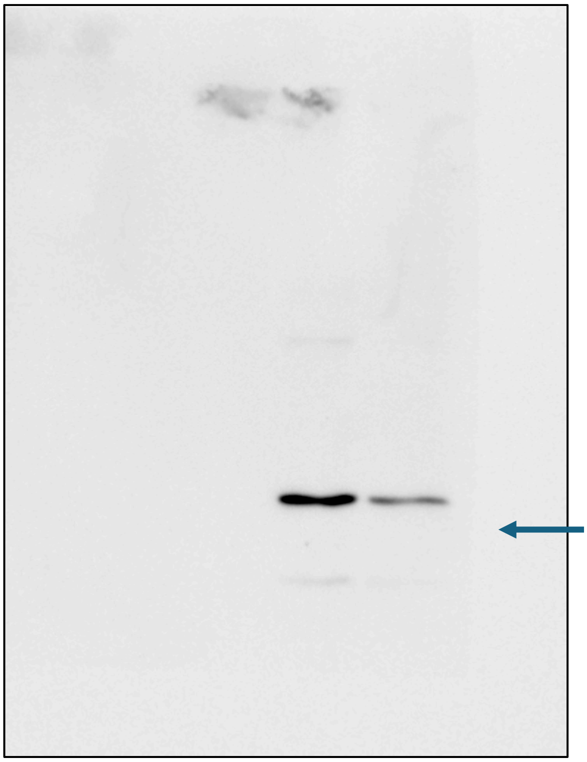

Cleaved Caspase 3

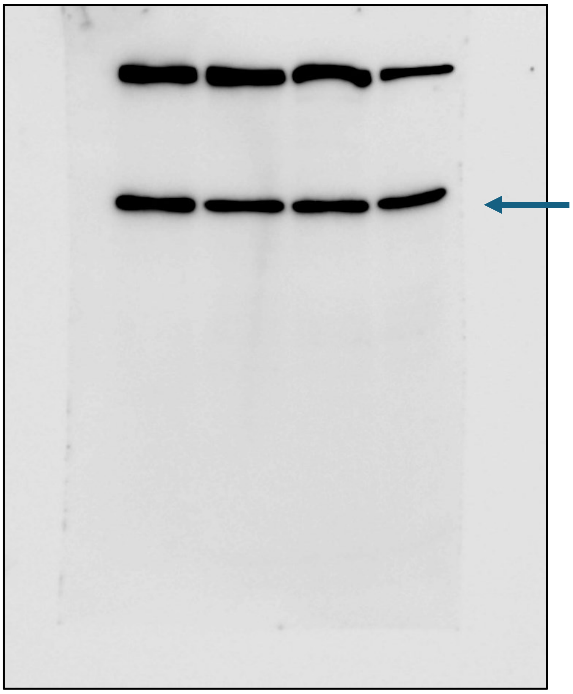

Tubulin

Fig. 6F H2O2

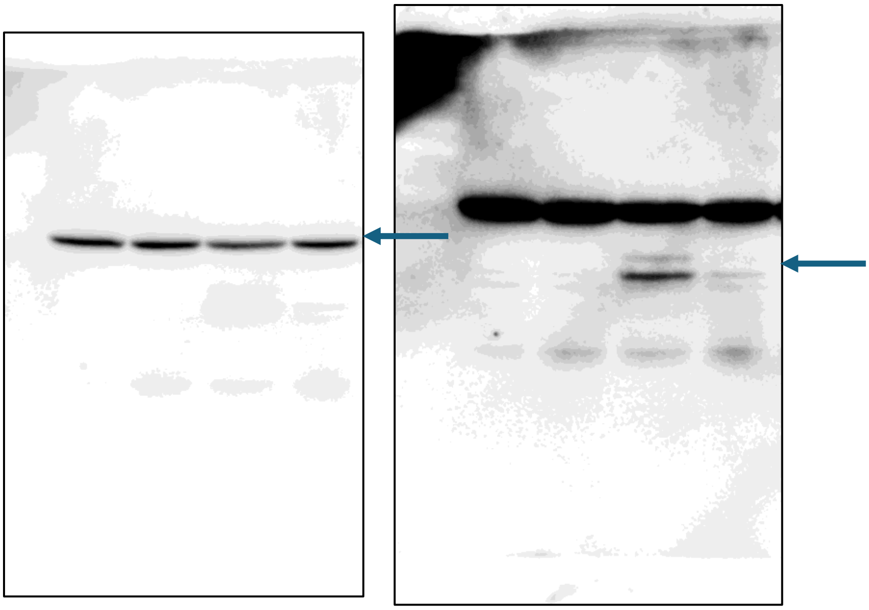

Caspase 9

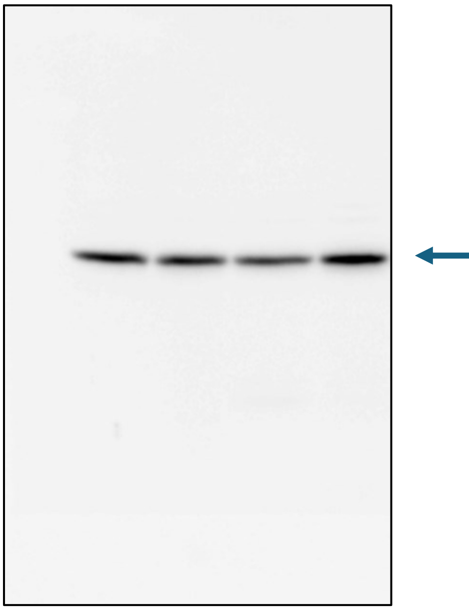

Caspase 3

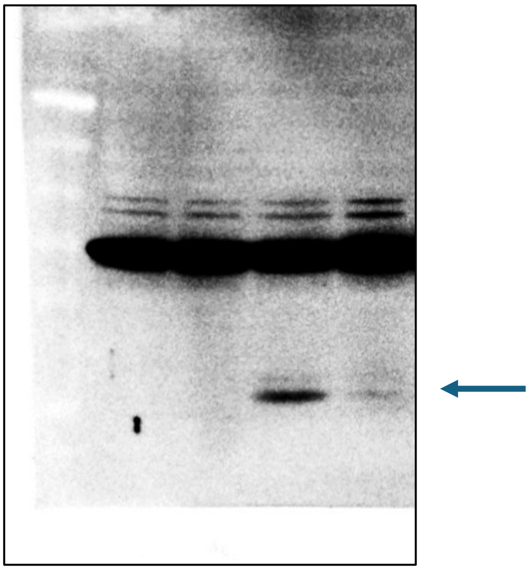

Caspase 3

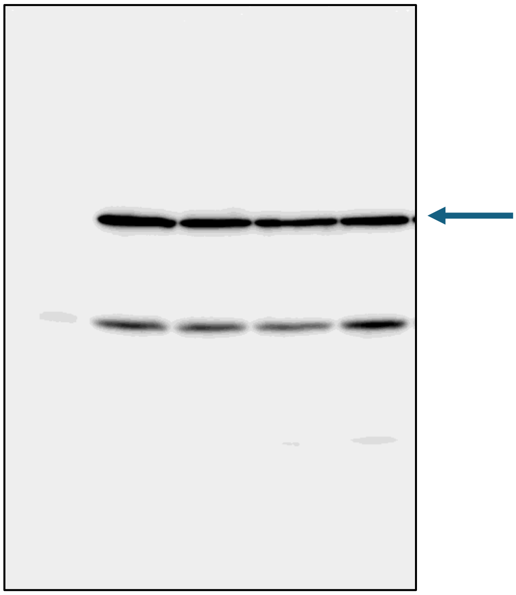

Tubulin

Fig. 6I

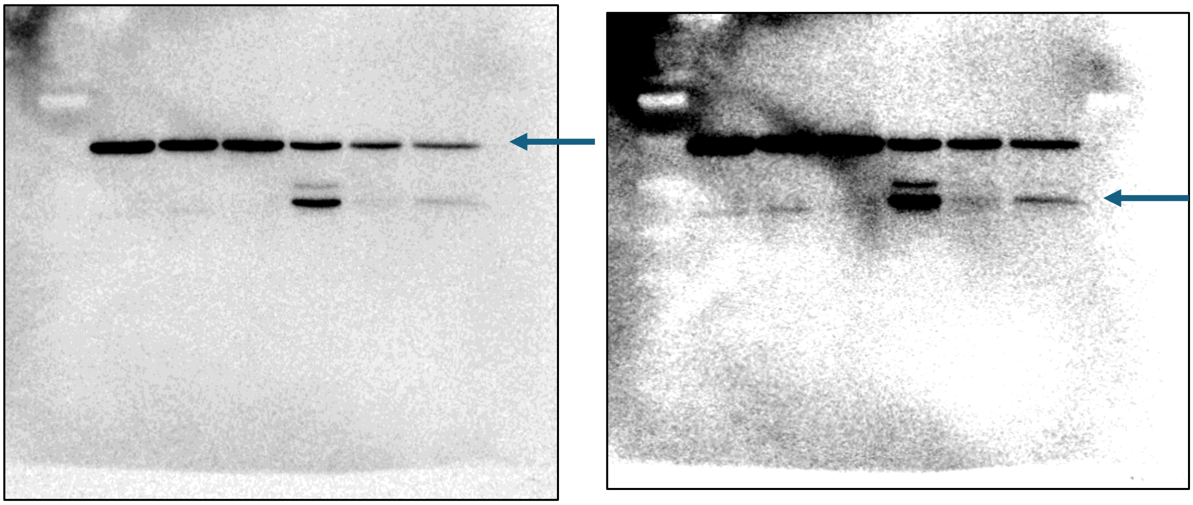

Caspase 9

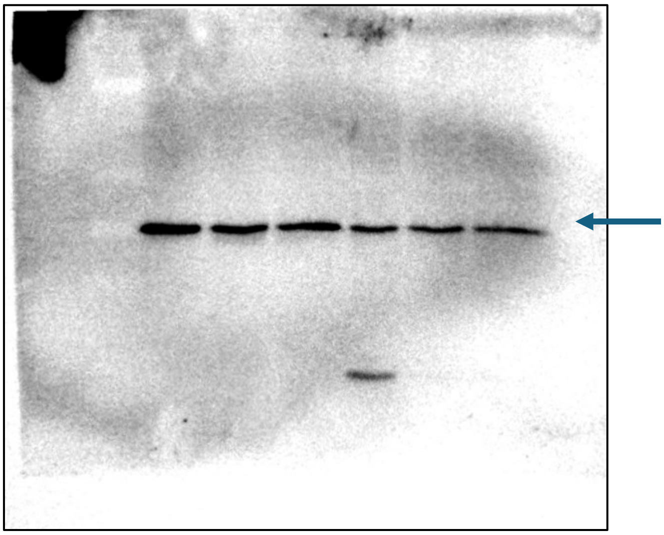

Caspase 3

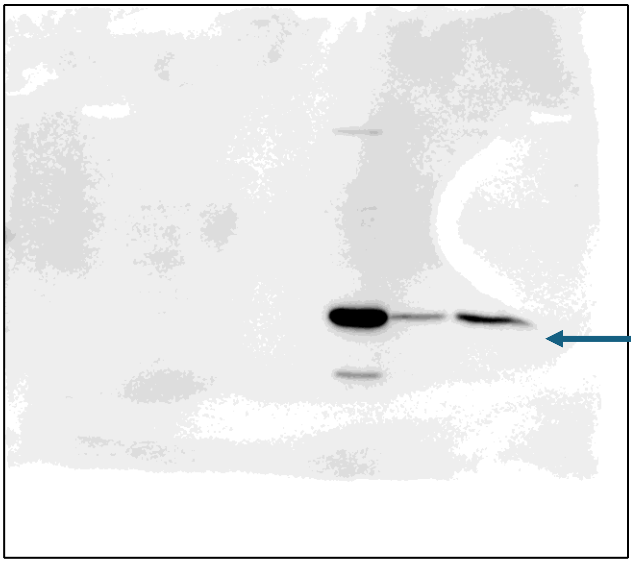

Cleaved Caspase 3

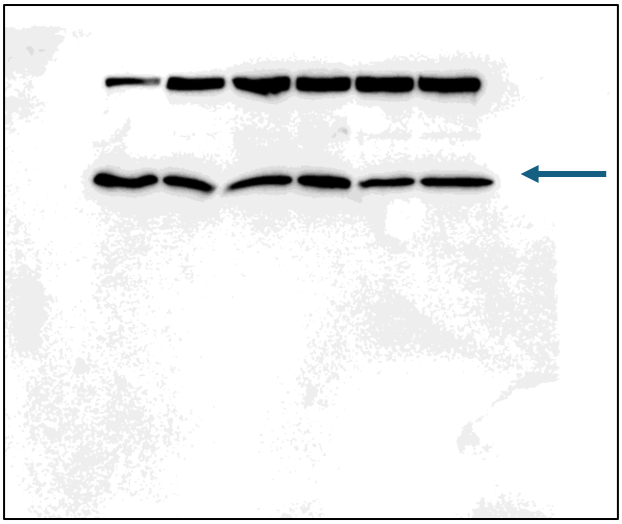

Tubulin

Fig. S1B

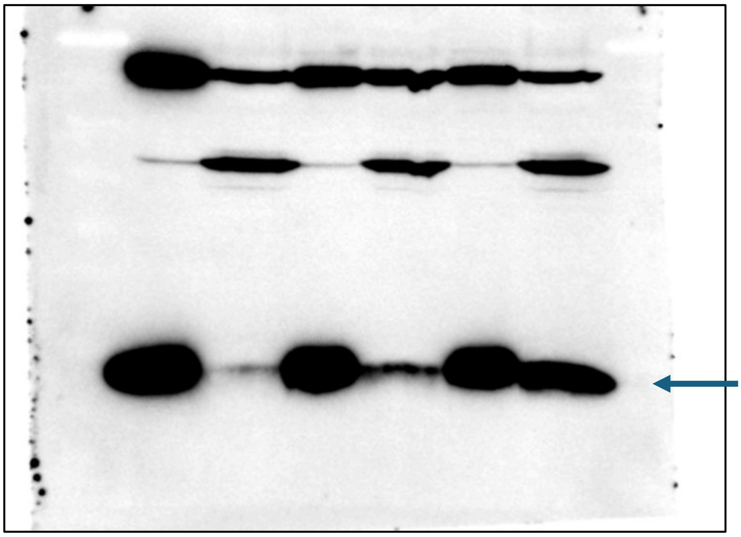

Cyt.c

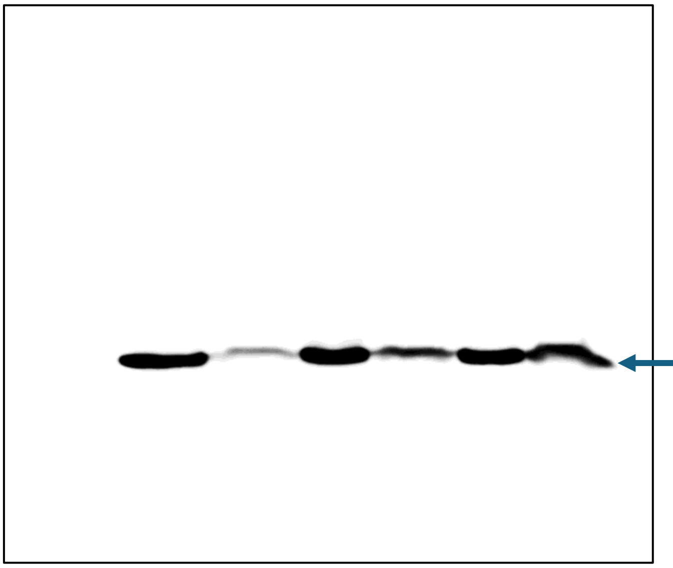

SMAC

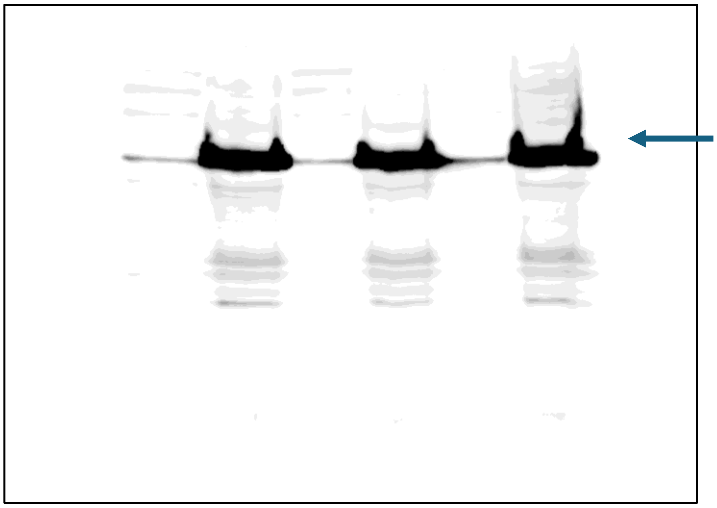

Tubulin

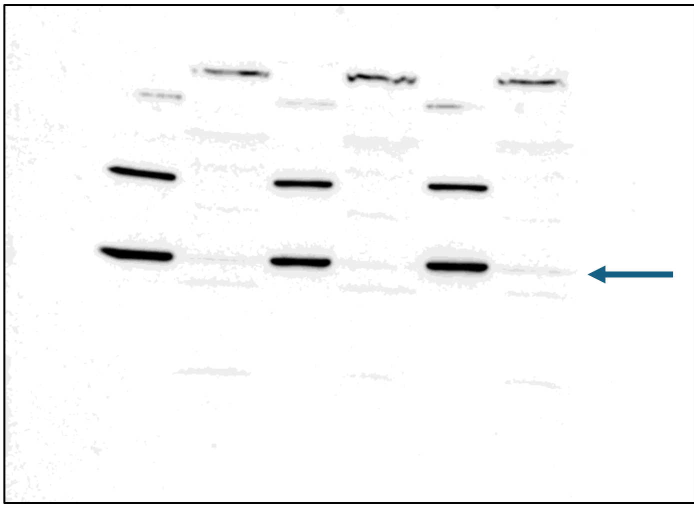

VDAC

Fig. S1C

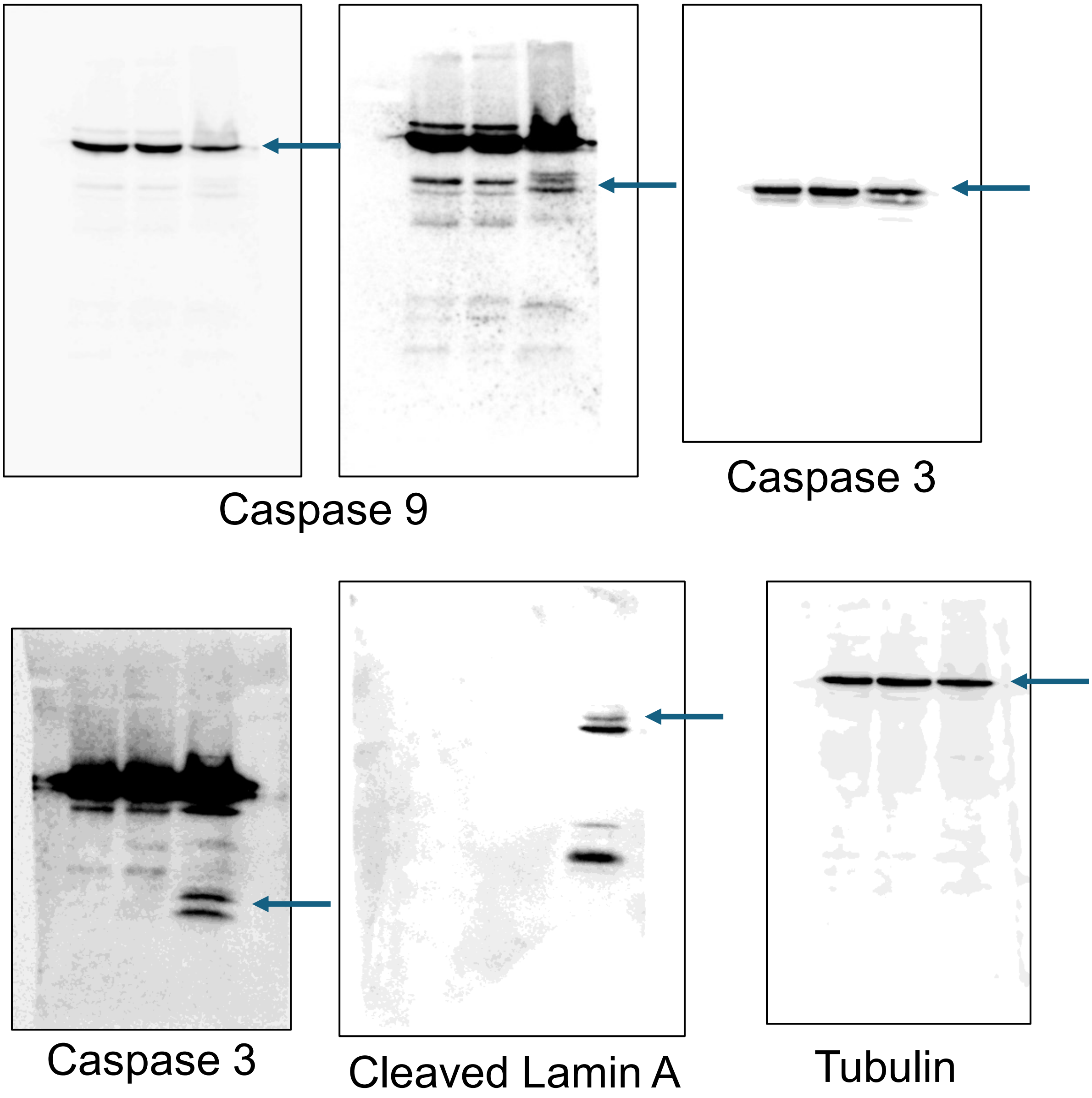

Fig. S2A

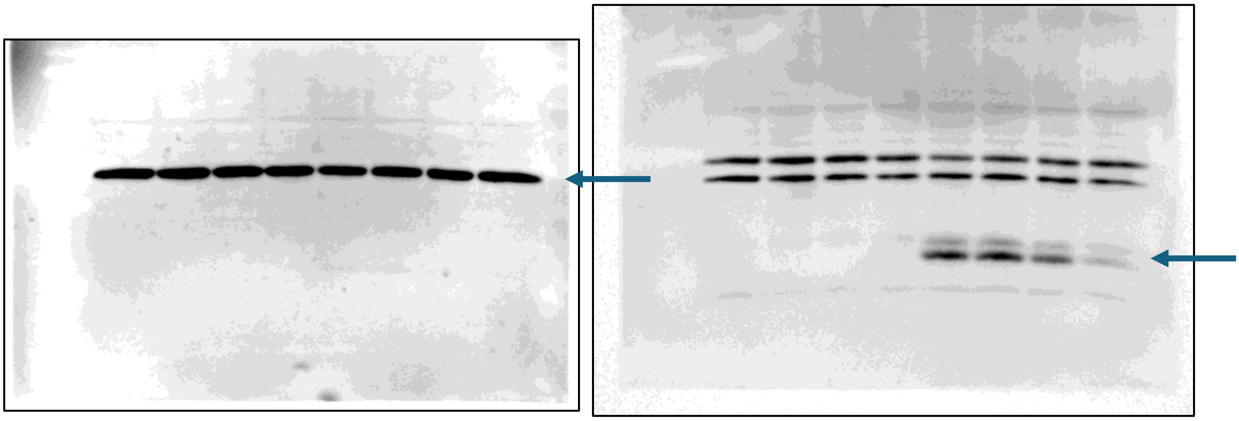

Caspase 3

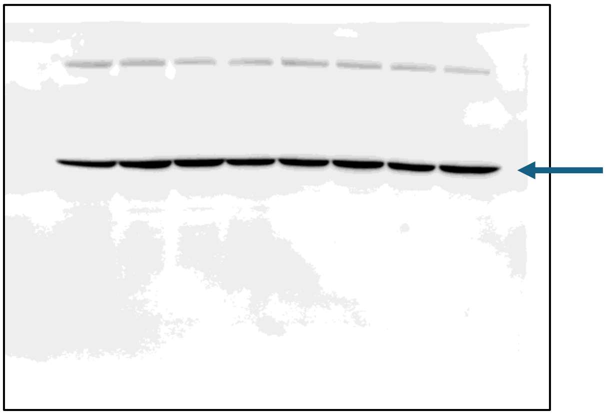

Tubulin

Fig. S2D

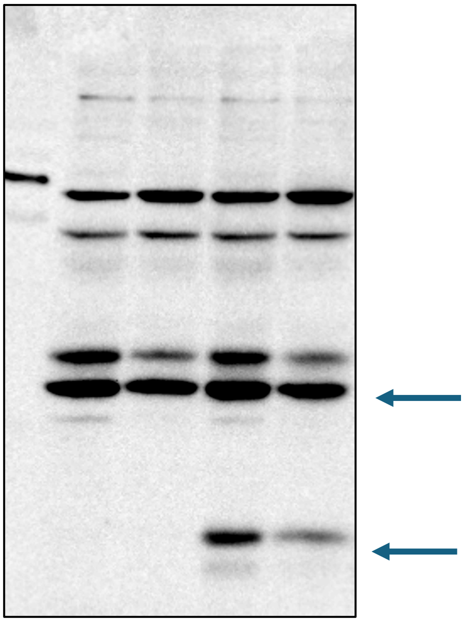

Caspase 3

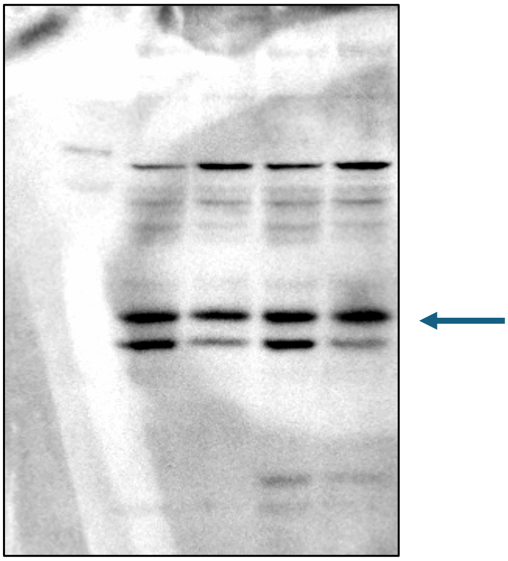

VDAC

Fig. S3A

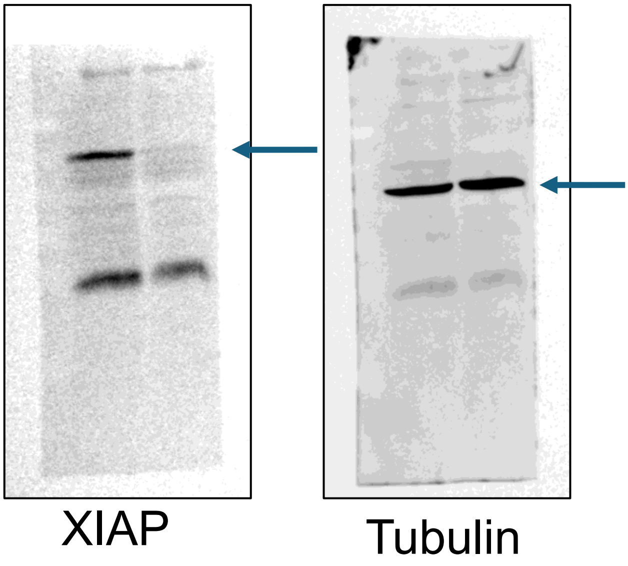

Fig. S4C

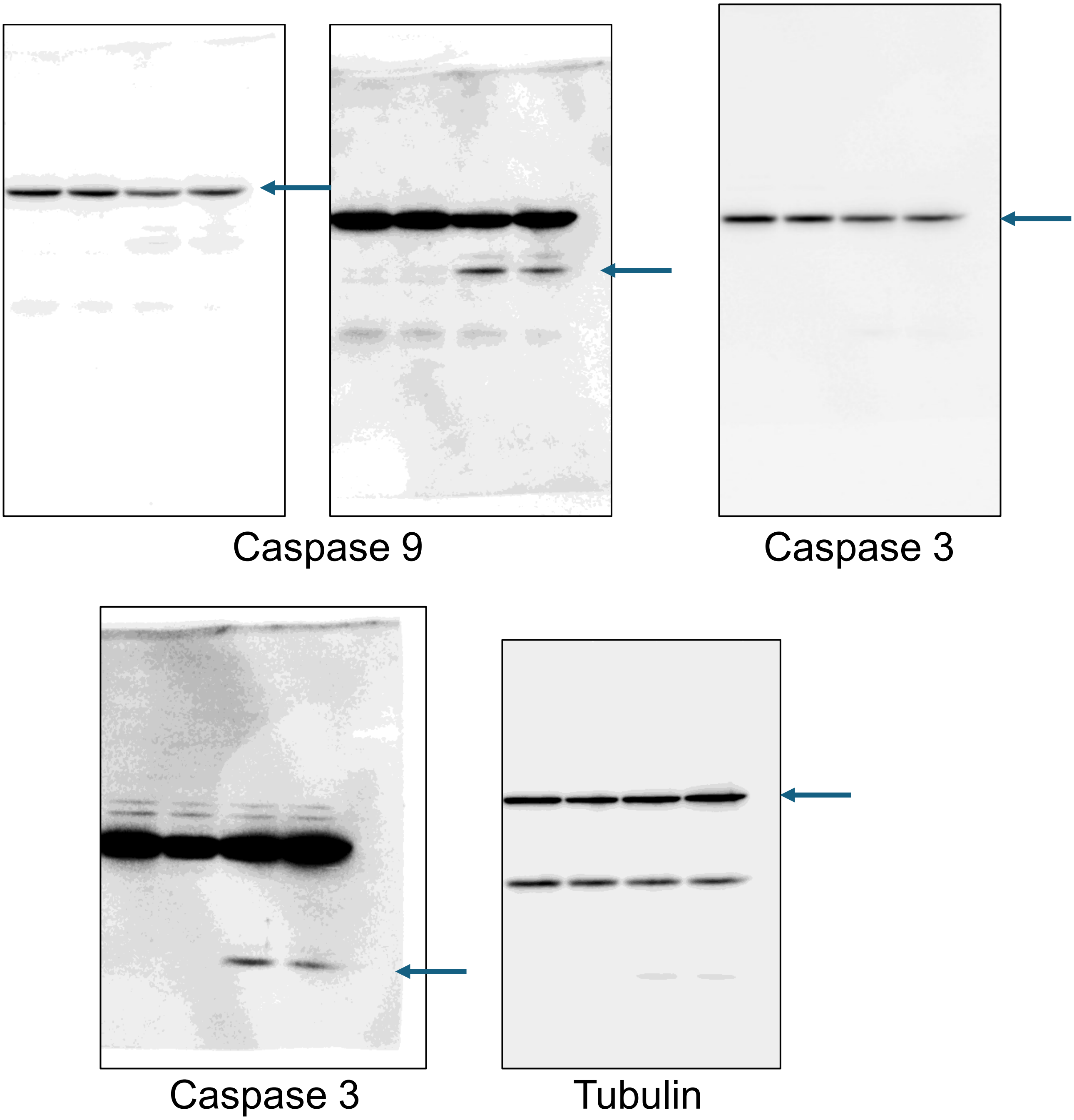

Supplement: Supplementary file 4 — uncropped Western Blots [file 41419_2024_6998_MOESM4_ESM.pdf]
